# Transcription factor binding site clusters identify target genes with similar tissue-wide expression and buffer against mutations

**DOI:** 10.12688/f1000research.17363.2

**Published:** 2019-04-08

**Authors:** Ruipeng Lu, Peter K. Rogan

**Affiliations:** 1 Computer Science, University of Western Ontario, London, Ontario, N6A 5B7, Canada; 2 Biochemistry, University of Western Ontario, London, Ontario, N6A 5C1, Canada; 3 Cytognomix, London, Ontario, N5X 3X5, Canada

**Keywords:** Transcription factors, position-specific scoring matrices, chromatin, binding sites, gene expression profiles, Bray-Curtis similarity, mutation, machine learning, information theory

## Abstract

**Background: **The distribution and composition of *cis*-regulatory modules composed of transcription factor (TF) binding site (TFBS) clusters in promoters substantially determine gene expression patterns and TF targets. TF knockdown experiments have revealed that TF binding profiles and gene expression levels are correlated. We use TFBS features within accessible promoter intervals to predict genes with similar tissue-wide expression patterns and TF targets using Machine Learning (ML).

**Methods: **Bray-Curtis Similarity was used to identify genes with correlated expression patterns across 53 tissues. TF targets from knockdown experiments were also analyzed by this approach to set up the ML framework. TFBSs were selected within DNase I-accessible intervals of corresponding promoter sequences using information theory-based position weight matrices (iPWMs) for each TF. Features from information-dense clusters of TFBSs were input to ML classifiers which predict these gene targets along with their accuracy, specificity and sensitivity. Mutations in TFBSs were analyzed *in silico* to examine their impact on TFBS clustering and predict changes in gene regulation.

**Results:** The glucocorticoid receptor gene ( *NR3C1*), whose regulation has been extensively studied, was selected to test this approach. *SLC25A32 *and *TANK *exhibited the most similar expression patterns to *NR3C1*. A Decision Tree classifier exhibited the best performance in detecting such genes, based on Area Under the Receiver Operating Characteristic curve (ROC). TF target gene prediction was confirmed using siRNA knockdown, which was more accurate than CRISPR/CAS9 inactivation. TFBS mutation analyses revealed that accurate target gene prediction required  at least 1  information-dense TFBS cluster.

**Conclusions**: ML based on TFBS information density, organization, and chromatin accessibility accurately identifies gene targets with comparable tissue-wide expression patterns. Multiple information-dense TFBS clusters in promoters appear to protect promoters from effects of deleterious binding site mutations in a single TFBS that would otherwise alter regulation of these genes.

## Introduction

The distinctive organization and combination of TFBSs and regulatory modules in promoters dictates specific expression patterns within a set of genes ^1^. Clustering of multiple adjacent binding sites for the same TF (homotypic clusters) and for different TFs (heterotypic clusters) defines *cis*-regulatory modules (CRMs) in human gene promoters. Experimental studies have shown that these clusters can reinforce (and in some instances, amplify) the impact of individual TFBSs on gene expression through increasing binding affinities, facilitated diffusion mechanisms and funnel effects ^2^. Because tissue-specific TF-TF interactions in TFBS clusters are prevalent, these features can assist in identifying correct target genes by altering binding specificities of individual TFs ^3^. Previously, we derived iPWMs from ChIP-seq data that can accurately detect TFBSs and quantify their strengths by determining the associated *R _i_* values (Rate of Shannon information transmission for an individual sequence ^4^). *R _sequence_* (the area under the sequence logo) is the average of *R _i_* values of all binding site sequences and represents the average binding strength of the TF ^3^. Neighboring, likely coregulatory TFBSs were identified by information density-based clustering (IDBC), which takes into account both the spatial organization (i.e. intersite distances) and information density (i.e. *R _i_* values) of TFBSs ^5^.

TF binding profiles, either derived from *in vivo* ChIP-seq peaks ^6– 8^ or computationally detected binding sites and CRMs ^9^, have been shown to be predictive of absolute gene expression levels using a variety of tissue-specific ML classifiers and regression models. Because signal strengths of ChIP-seq peaks are not strictly proportional to strengths of the contained strongest TFBSs and are instead controlled by TFBS counts ^3, 10^, representing TF binding strengths by ChIP-seq signals may not be appropriate; nevertheless, both achieved similar accuracy ^11^. CRMs have been formed by combining two or three adjacent TFBSs ^9^, which is inflexible, as it arbitrarily limits the number of binding sites contained in a module, and does not consider differences between information densities of different CRMs. Chromatin structure (e.g. histone modification (HM) and DNase I hypersensitive sites (DHSs)) were also found to be statistically redundant with TF binding in explaining tissue-specific mRNA transcript abundance at a genome-wide level ^7, 8, 12, 13^, which was attributed to the heterogeneous distribution of HMs across chromatin domains ^8^. Combining these two types of data explained the largest fraction of variance in gene expression levels in multiple cell lines ^7, 8^, suggesting that either contributes unique information to gene expression that cannot be compensated for by the other.

Previous studies have shown that a small subset of target genes bound by TFs were differentially expressed (DE) in the GM19238 cell line, upon knockdown with small interfering RNAs (siRNAs) ^14^. TFBS counts were defined as the number of ChIP-seq peaks overlapping the promoter, with the caveat that the number and strengths of the TFBSs in each peak were not known ^15^. Correlation between total TFBS counts and gene expression levels across 10 different cell lines was more predictive of which were DE than by setting a minimum threshold count of TFBSs ^15^. This has also been addressed by perturbing gene expression with CAS9-directed clustered regularly interspaced short palindromic repeats (CRISPR) of 10 different TF genes in K562 cells ^16^. The regulatory effects of each TF were dissected by single cell RNA sequencing with a regularized linear computational model ^16^ . This revealed DE targets and new functions of individual TFs, some of which were likely regulated through direct interactions at TFBSs in their corresponding promoters. ML classifiers have also been applied to predict targets of a single TF using features extracted from *n*-grams derived from consensus binding sequences ^17^, or from TFBSs and homotypic binding site clusters ^5^.

We investigated whether the predicted TFBS strengths, distribution and CRM composition in promoters substantially determines gene expression profiles of direct TF targets. A general ML framework was developed by combining information theory-based TF binding profiles with DHSs. The approach predicts which genes have similar tissue-wide expression profiles, and conversely, DE direct TF targets. Upon selecting DHSs to define accessible promoter intervals, ML features that captured the spatial distribution and information theory-based TFBS compositions of CRMs were extracted from IDBC clusters. The intent of this framework was to provide insight into the transcriptional program of genes with similar profiles by dissecting shared properties of their *cis*-regulatory element organization, without imposing strict constraints on the strengths and distributions of TFBSs. We first identify genes with comparable tissue-wide expression profiles by application of Bray-Curtis similarity ^18^. Using transcriptome data generated by CRISPR- ^16^ and siRNA-based ^14^ TF knockdowns, we predicted DE target genes with promoters that contain ChIP-seq peaks for these same TFs.

## Methods

To identify genes with similar tissue-wide expression patterns, we formally define tissue-wide gene expression profiles and pairwise similarity measures between profiles of different genes. A general ML framework relates features extracted from the organization of TFBSs in these genes to their tissue-wide expression patterns. Since protein-coding (PC) sequences represent the most widely studied and best understood component of the human genome ^19^, positives and negatives for deriving ML classifiers of predicted DE direct TF target genes encoding proteins (TF targets, below) were obtained from CRISPR- and siRNA-generated knockdown data.

### Similarity between GTEx tissue-wide expression profiles of genes

We used data from the Genotype-Tissue Expression (GTEx, version 6p) project which measured expression levels for each gene in 53 tissues, in different numbers of individuals (5 to 564). For each tissue population, the median expression value is given in RPKM (Reads Per Kilobase of transcript per Million mapped reads) for each gene ^20^. Data are available on Zenodo ^21^. To capture the tissue-wide overall expression pattern of a gene instead of within a single tissue, the tissue-wide expression profile of a gene was defined as its median RPKM across GTEx tissues, which is described by a 53-element vector ( Equation 1). Note that different isoforms whose expression patterns may significantly differ from each other cannot be distinguished by this approach.

$$EP^{A}=[MEV_{1}^{A},MEV_{\text{2}}^{A},\cdots ,MEV_{\text{53}}^{A}]\;(in\;RPKM)\;(1)$$

where *EP ^A^* is the tissue-wide expression profile of Gene *A*, $MEV_{\text{1}}^{A}$ is the median expression value of Gene *A* in Tissue 1, $MEV_{\text{2}}^{A}$ is the median expression value of Gene *A* in Tissue 2, etc.

To discover other genes whose tissue-wide expression profiles are similar to a given gene, we computed the Bray-Curtis Similarity ( Equation 2) between the tissue-wide expression profiles of all gene pairs. Relative to other similarity measures ( Table 1, Additional file 1 ^22^), this function exhibits desirable properties, including: 1) being bounded between 0 and 1, 2) achieving maximal similarity of 1, if and only if two vectors are identical, and 3) larger values having a larger impact on the resultant similarity value.

**Table 1.** Comparison between metrics in measurement of similarity between GTEx tissue-wide expression profiles of genes.

Similarity metric	Property 1 †, ‡	Property 2 †	Property 3 †
Bray-Curtis	√; [0,1]	√	√
Euclidean	√; (0,1]	√	×
Cosine	√; [0,1]	×	√
Pearson correlation ^25^	×; [-1,1]	×	×
Spearman correlation ^26^	×; [-1,1]	×	×

†The symbols √ and ×, respectively, indicate that the similarity metric satisfies and does not satisfy the property. ‡The interval in each cell indicates the range in which the result computed by the similarity metric lies.

$$sim_{Bray-Curtis}(EP^{A},EP^{B})=\left\{\begin{array}{l} 1,\;if\;\sum_{i=1}^{\text{53}}MEV_{i}^{A}=\;\sum_{i=\text{1}}^{\text{53}}MEV_{i}^{B}=0\\ 1-\frac{\sum_{i=\text{1}}^{\text{53}}\left|MEV_{i}^{A}-MEV_{i}^{B}\right|}{\sum_{i=1}^{53}\left(MEV_{i}^{A}+MEV_{i}^{B}\right)},\;otherwise \end{array}\;(2\right)$$

### Prediction of genes with similar tissue-wide expression profiles

**Figure 1.**
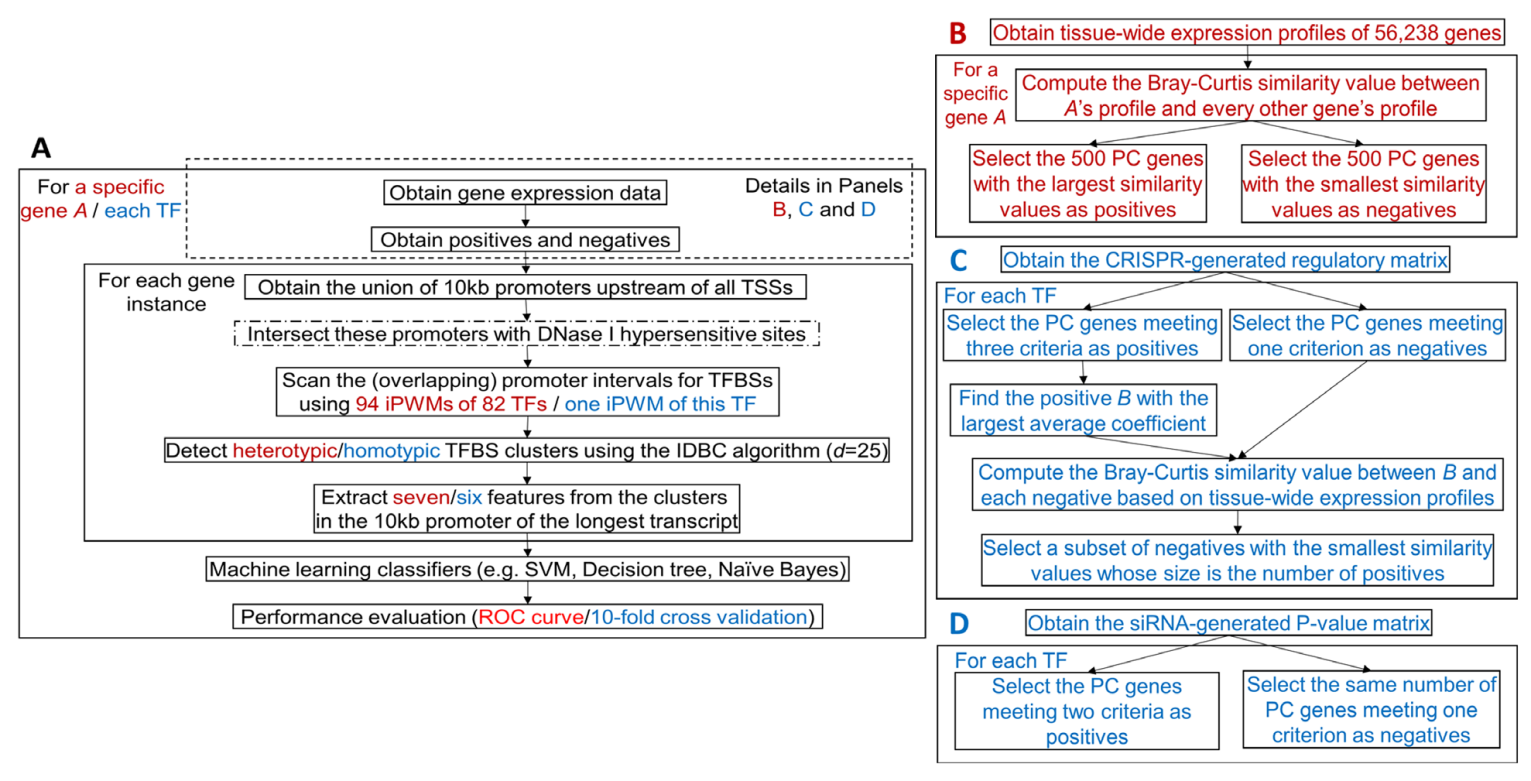
The general framework for predicting genes with similar tissue-wide expression profiles and TF targets. Red and blue contents are respectively specific to prediction of genes with similar tissue-wide expression profiles and prediction of TF targets. ( **A**) An overview of the ML framework. The steps enclosed in the dashed rectangle vary across prediction of genes with similar tissue-wide expression profiles and TF targets. The step with a dash-dotted border that intersects promoters with DHSs is a variant of the primary approach. In the IDBC algorithm (Additional file 1 ^22^), the parameter *I* is the minimum threshold on the total information contents of TFBS clusters. In prediction of genes with similar tissue-wide expression profiles, the minimum value was 939, which was the sum of mean information contents ( *R _sequence_* values) of all 94 iPWMs; in prediction of direct targets, this value was the *R _sequence_* value of the single iPWM used to detect TFBSs. The parameter *d* is the radius of initial clusters in base pairs, whose value, 25, was determined empirically. The seven ML features derived from TFBS clusters are described in the Methods section. The performance of seven different classifiers was evaluated with ROC curves and 10-fold cross validation (Additional file 1 ^22^). ( **B**) Obtaining the positives and negatives for identifying genes with similar tissue-wide expression profiles to a given gene (Additional file 2 ^22^). ( **C**) Obtaining the positives and negatives for predicting target genes of seven TFs using the CRISPR-generated perturbation data in K562 cells (Additional file 3 ^22^). ( **D**) Obtaining the positives and negatives for predicting target genes of 11 TFs using the siRNA-generated knockdown data in GM19238 cells (Additional file 4 ^22^).

The framework for predicting whether the tissue-wide expression profile of a gene resembles a particular gene is indicated in Figure 1 (panels A and B). The genomic locations of all DHSs in 95 cell types from the ENCODE project[ 23; hg38 assembly] were filtered for known promoters ^24^; these sequences were then scanned for TFBSs using 94 iPWMs corresponding to the primary binding motifs for 82 TFs ^3^. Data are available on Zenodo ^21^. Heterotypic TFBS clusters were detected with the IDBC algorithm by specifying a minimum threshold of 0.1* *R _sequence_* for the *R _i_* values of individual TFBS elements in potential clusters; this eliminated weak binding sites detected with iPWMs corresponding to false positive, non-functional TFBSs ^3^.

The seven information density-related ML features (Additional file 1 ^22^) derived from each TFBS cluster included: 1) The distance between this cluster and the transcription start site (TSS), 2) The length of this cluster, 3) The information content of this cluster (i.e. the sum of *R _i_* values of all TFBSs in this cluster), 4) The number of binding sites of each TF within this cluster, 5) The number of strong binding sites ( *R _i_ > R _sequence_*) of each TF within this cluster, 6) The sum of *R _i_* values of binding sites of each TF within this cluster, and 7) The sum of *R _i_* values of strong binding sites ( *R _i_ > R _sequence_*) of each TF within this cluster.

Each of the Features 1–3 was defined in a gene as a vector, whose size equalled the number of clusters in the gene promoter; each cluster was mapped to a single value in the vector. In Features 4–7, each cluster itself was mapped to a vector corresponding to binding sites for 82 TFs (Additional file 1 ^22^). If two genes contained different numbers of clusters, the maximum number of clusters among all instances was determined, and null clusters were added to the 5’ end of promoters with the smaller numbers of clusters; this enabled all genes to exhibit the same cluster counts. This allowed all genes to be used as training data for ML classifiers. Default parameter values for these classifiers were used to generate ROC curves with a built-in MATLAB function (Additional file 1 ^22^).

### Prediction of TF targets

***Perturbed target gene expression after CRISPR-based mutation of TF genes.*** Dixit *et al*. performed CRISPR-based perturbation experiments using multiple guide RNAs for each of ten TFs in K562 cells, resulting in a matrix of coefficients indicating the effect of each guide RNA on each of 22,046 genes ^16^. Data are available on Zenodo ^21^. The coefficient of a guide RNA is defined as the log _10_ fold change in gene expression level ^16^. We previously derived iPWMs for the primary binding motifs of 7 of the ten TFs (EGR1, ELF1, ELK1, ETS1, GABPA, IRF1, YY1) ^3^. The framework for predicting TF targets ( Figure 1A and 1C) was applied to these TFs. We defined a positive TF target gene in a cell line as :

1) The fold change in the expression level of this gene for each of the guide RNAs of the TF was consistently greater than (or is less than) 1, which eliminated genes exhibiting both increased and decreased expression levels for different guide RNAs, and increased the possibility that the gene was downregulated (or upregulated) by the TF (Additional file 1 ^22^). and
2) The average fold change in the expression level of this gene for all guide RNAs of the TF was greater than the threshold (or is less than 1/), and
3) The promoter interval (10 kb) upstream of a TSS of this gene overlapped a ChIP-seq peak of the TF in the cell line.

If the coefficients for all guide RNAs of the TF for a gene were zero, the gene was defined as a negative (i.e. a non-target gene). As the threshold *ε* increases, the number of positives strictly decreases. As *ε* decreases, we have lower confidence that the positives were DE as a result of the TF perturbation. We balanced the number of positives obtained against our confidence that they were DE by evaluating different values of *ε* (i.e. 1.01, 1.05 and 1.1; Additional file 1 ^22^). For each TF, all ENCODE ChIP-seq peak datasets from the K562 cell line were merged to determine positives. Data are available on Zenodo ^21^. To avoid imbalanced datasets that significantly compromised the classifier performance ^27^, the Bray-Curtis function was applied to compute the similarity values in the tissue-wide expression profile between all negatives and the positive gene with the largest average coefficient, and then negatives with the smallest values were selected, resulting in the same number of positive and negative genes ( Figure 1C).

Because TFs may exhibit tissue-specific sequence preferences due to different sets of target genes ^3^, the K562 cell line-derived iPWMs of EGR1, ELK1, ELF1, GABPA, IRF1, YY1 were used to detect binding sites in DHS-intersected intervals; for ETS1, we used the only available iPWM from GM12878 ^3^. Six features (Features 1-5 and 7) were derived from each homotypic cluster (i.e. Feature 6 became identical to Feature 3, because only binding sites from a single TF were used) ( Figure 1A). The results of 10 rounds of 10-fold cross validation were averaged to evaluate the accuracy of the classifier.

***Target gene expression changes after siRNA-based knockdown of TFs.*** Significant changes in expression of target genes upon knockdown of 59 TFs in the GM19238 line were identified from the probability of differences in expression level (P-value) relative to the null hypothesis of no change ^14^. Data are available on Zenodo ^21^. DE genes with larger changes exhibited smaller P-values. The distribution of ChIP-seq peaks were considered to be evidence of TF binding to the promoters of these genes ^14^. Among these 59 TFs, we have previously derived iPWMs exhibiting primary binding motifs for 11 (BATF, JUND, NFE2L1, PAX5, POU2F2, RELA, RXRA, SP1, TCF12, USF1, YY1) ^3^. For this reason, transcription targets of these TFs were predicted in the GM19238 cell line ( Figure 1A, D).

For ML training, we defined a positive (i.e. a target gene) for a TF, if the P-value of this gene was ≤ 0.01, and the promoter interval up to 10 kb upstream of a TSS overlapped one or more ChIP-seq peaks of the TF in GM12878. Other genes with P-values > 0.01 exhibited insufficient support for being TF targets and were labeled as negatives.

The iPWMs from GM19238 were used to detect binding sites for all TFs except for RELA, RXRA and NFE2L1. The iPWM from the GM19099 line was used for RELA, and for RXRA and NFE2L1, the only available iPWMs were derived from HepG2 and K562 cells, respectively. The DHSs in GM19238 were first remapped to the hg38 assembly using liftOver, prior to being intersected with known promoters ^28^. Data are available on Zenodo ^21^. Although the knockdowns were performed in GM19238, GM12878 and GM19099 are also lymphoblastic cell lines, with GM19099 and GM19238 both being derived from the Yoruban population. For this analysis, the iPWMs derived in GM12878 and GM19099 were more appropriate sources of accessible TFBSs than those from HepG2 and K562, since GM12878 and GM19099 are more likely comparable to GM19238 than HepG2 and K562. ML results were evaluated by averaging cross validation, as described above.

### Mutation analyses on promoters of TF targets

To understand the significance of individual binding sites on the regulatory state of TF targets, we evaluated the effects of sequence changes in TFBSs that altered the *R _i_* values of these sites, the definition of TF clusters, and whether consequential IDBC-related changes impacted the prediction of TF target genes. For example, mutations were sequentially introduced into the strongest binding sites in TFBS clusters of the EGR1 target gene, *MCM7*, to determine the threshold for cluster formation and whether disappearing clusters disabled *MCM7* expression. For one target gene of each TF from the CRISPR-generated perturbation data, effects of naturally occurring TFBS variants present in dbSNP ^29^ were also evaluated to explore aspects of TFBS organization that enabled both clusters and promoter activity to be resilient to binding site mutations. This was done by analyzing whether the occurrence of individual or multiple single nucleotide polymorphisms (SNPs) lead to the loss of binding sites and the corresponding clusters, and resulted in changes in the predictions for these targets.

## Results

### The Bray-Curtis Function can accurately quantify the similarity between tissue-wide gene expression profiles

**Figure 2.**
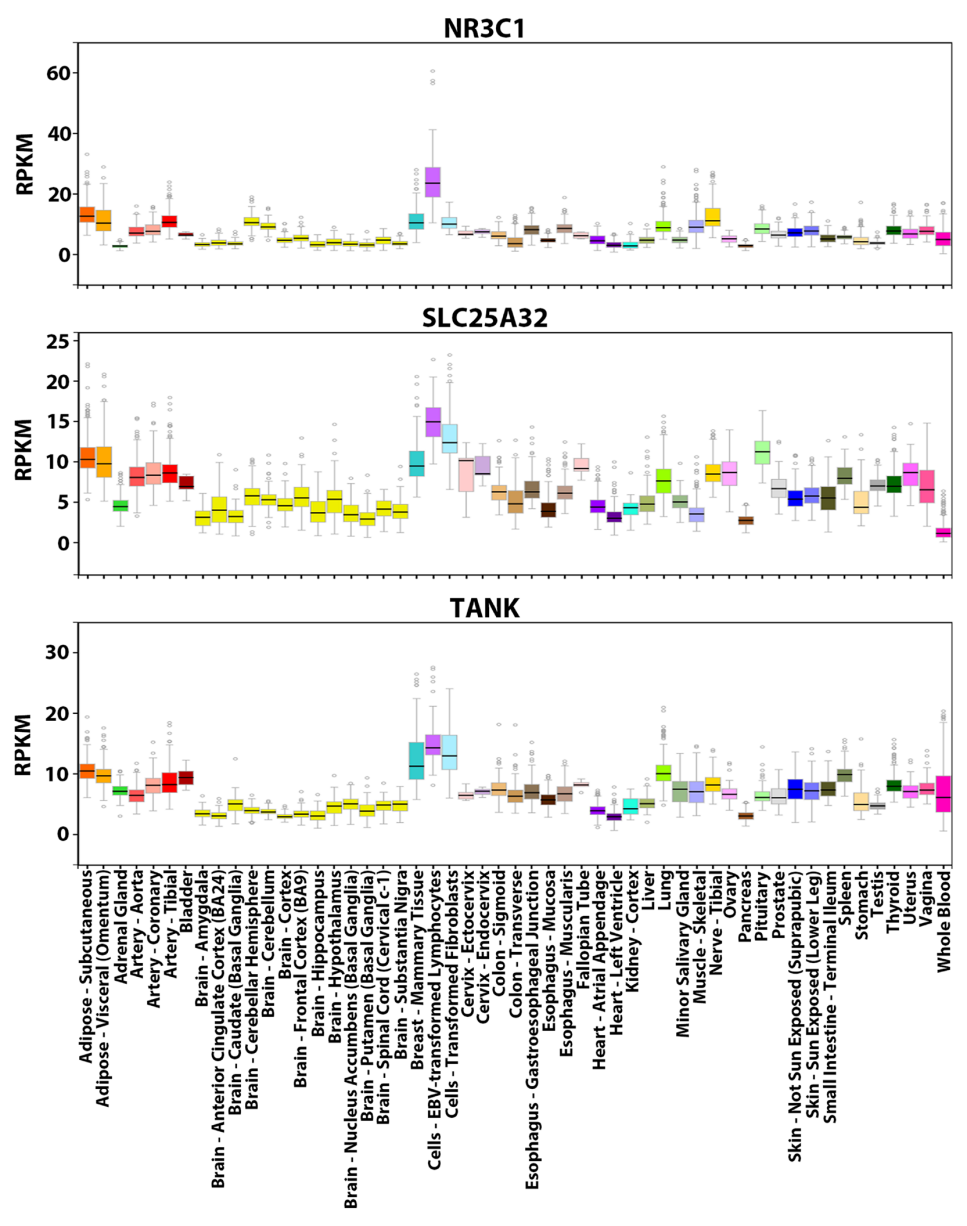
GTEx tissue-wide expression profiles of *NR3C1*, *SLC25A32* and *TANK*. Visualization of the expression values (in RPKM) of these genes across 53 tissues from GTEx. For each gene, the colored rectangle belonging to each tissue indicates the valid RPKM of all samples in the tissue, the black horizontal bar in the rectangle indicates the median RPKM, the hollow circles indicate the RPKM of the samples considered as outliers, and the grey vertical bar indicates the sampling error. A comparison of the panels shows that the overall expression patterns of the three genes across the 53 tissues resemble each other (e.g. all three genes exhibit the highest expression levels in lymphocytes and the lowest levels in brain tissues).

We computed the similarity values ( Equation 2) between the tissue-wide expression profiles of the glucocorticoid receptor ( *GR* or *NR3C1*) gene and all other 18,812 PC genes to find genes with related profiles. NR3C1 is an extensively characterized TF with many known direct target genes ^30^. As a constitutively expressed TF activated by glucocorticoid ligands, the protein mediates up-regulation of anti-inflammatory genes by binding as homodimers to glucocorticoid response elements. Transcription of proinflammatory genes is down-regulated by complexing with other activating TFs (e.g. NFKB and AP1), thereby eliminating their ability to bind and activate targets ^30^. NR3C1 can bind its own promoter forming an auto-regulatory loop, which also contains functional binding sites of 11 other TFs (e.g. SP1, YY1, IRF1, NFKB) whose iPWMs have been developed and/or mutual interactions have been described previously ^3, 30^. The tissue-wide expression profile of NR3C1 comprises all different splicing and translational isoforms ( *GRα-A*, *GRα-B*, *GRα-C*, *GRα-D*, *GRβ*, *GRγ*, *GRδ*). However, the profile averages out tissue-specific preferences of some isoforms, for example, *GRα-C* isoforms are significantly higher in the pancreas and colon, whereas levels of *GRα-D* are highest in spleen and lungs ^30^. We found that *SLC25A32* and *TANK* have the greatest similarity in expression to *NR3C1* (0.880 and 0.877 respectively), based on their overall similar expression patterns across the 53 tissues ( Figure 2).

### The Decision Tree classifier performed best in prediction of genes with similar tissue-wide expression profiles

**Figure 3.**
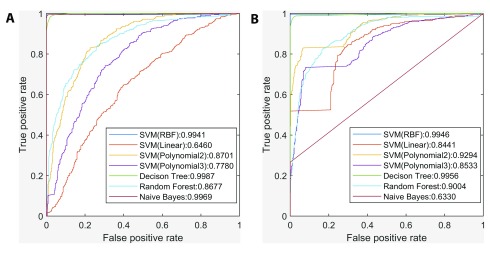
Comparison between the performance of different classifiers in prediction of genes with similar tissue-wide expression profiles to *NR3C1*. ( **A**) ROC curves and AUC of seven classifiers without intersecting promoters with DHSs. ( **B**) ROC curves and AUC of seven classifiers after intersecting promoters with DHSs. The Decision Tree classifier exhibited the largest AUC under both scenarios, and inclusion of DHS information significantly improved other classifiers’ AUC except for Naïve Bayes.

Several ML classifiers (Naïve Bayes, Decision Tree (DT), Random Forest and Support Vector Machines (SVM) with four different kernels) were evaluated to determine how well TFBS-related features could predict genes with tissue-wide expression profiles similar to *NR3C1*. Their performance were compared using ROC curves, for complete promoters or for accessible promoter sequences that were first intersected with DHSs ( Figure 3). DT exhibited the largest AUC (area under the curve) under both scenarios, and was one of two most stable classifiers (i.e. ΔAUC < 0.01), with the other being the SVM with RBF kernel. Consistent with previous findings ^10, 31, 32^, inclusion of DHS information significantly improved AUC values of the other classifiers with the exception of Naïve Bayes. In many instances, all TFBSs in a contiguous DHS interval formed a single binding site cluster.

### The Decision Tree classifier was predictive of TF target genes

Based on its performance in distinguishing genes with *NR3C1*-like tissue-wide expression profiles, the DT classifier was used to predict TF targets respectively based on the CRISPR- ^16^ and siRNA-generated ^14^ perturbation data. Performance was assessed with 10 rounds of 10-fold cross validation ( Tables 2 & Table 3). Gini importance values were also used to assess the relative contribution of the six ML features to the predictive power of the classifier (Additional file 5 ^22^). For the CRISPR-perturbed TFs in the K562 cell line, clustering Features 1-3, particularly the TFBS cluster information densities (Feature 3), were most important. The TFBS-level Feature 5 comprising the distribution of strong binding sites accounts for the largest contribution to classifier performance for the 11 siRNA-perturbed TFs in the GM19238 cell line. To assess the value of all ML features in capturing the distribution and composition of CRMs in promoters, all but one (TFBS counts) were sequentially removed, and the impact on accuracy of the classifier was determined. In each instance, classifier performance decreased, except for CRISPR-perturbed GABPA, IRF1 and YY1 upon inclusion of DHS information (Additional file 5 ^22^).

**Table 2.** The Decision Tree classifier performance for predicting TF targets using the CRISPR-generated knockdown data.

TF	Excluding DHS information †			Including DHS information †		
	Sensitivity	Specificity	Accuracy	Sensitivity	Specificity	Accuracy
EGR1	0.58	0.62	0.60	0.78	0.81	0.80
ELF1	0.59	0.65	0.62	0.83	0.87	0.85
ELK1	0.59	0.59	0.59	0.80	0.81	0.81
ETS1	0.59	0.6	0.59	0.81	0.81	0.81
GABPA	0.55	0.57	0.56	0.72	0.75	0.74
IRF1	0.54	0.55	0.54	0.76	0.64	0.70
YY1	0.50	0.51	0.51	0.45	0.69	0.57

†The average performance of 10 rounds of 10-fold cross validation when setting *ε* to 1.05 is indicated. The accuracy of each individual round is indicated in Additional file 5 ^22^. The CRISPR-generated knockdown data were obtained from Dixit *et al*. ^16^.

**Table 3.** The Decision Tree classifier performance for predicting TF targets using the siRNA-generated knockdown data.

TF	Excluding DHS information †			Including DHS information †		
	Sensitivity	Specificity	Accuracy	Sensitivity	Specificity	Accuracy
BATF	0.96	0.97	0.96	0.85	1	0.93
JUND	0.86	0.90	0.88	0.80	1	0.90
NFE2L1	0.92	0.95	0.94	0.71	0.93	0.82
PAX5	0.96	0.97	0.96	0.88	0.98	0.93
POU2F2	0.97	0.97	0.97	0.89	0.99	0.94
RELA	0.95	0.96	0.96	0.83	0.97	0.90
RXRA	0.93	0.91	0.92	0.84	0.95	0.89
SP1	0.98	0.98	0.98	0.89	0.99	0.94
TCF12	0.98	0.98	0.98	0.86	0.99	0.93
USF1	0.97	0.98	0.97	0.83	0.98	0.90
YY1	1	1	1	0.55	0.99	0.77

†The average performance of 10 rounds of 10-fold cross validation is indicated. The accuracy of each individual round is shown in Additional file 5 ^22^. The siRNA-generated knockdown data were obtained from Cusanovich *et al.* ^13^.

The DT classifier predicted TF targets with greater sensitivity and specificity, after eliminating TFBSs in inaccessible promoter intervals in the CRISPR-generated knockdown data ( Table 2). Specifically, predictions for EGR1, ELK1, ELF1, ETS1, GABPA, and IRF1 were more accurate than for YY1, which itself represses or activates a wide range of promoters by binding to sites overlapping the TSS ( Table 2). Accordingly, perturbation results showed that YY1 has ~4-22 fold more PC targets in the K562 cell line than the other TFs (ε = 1.05). Binding of YY1 more significantly impacts the expression levels of target genes. The ratio of the PC targets at ε = 1.1 vs ε = 1.01 was 0.334, which significantly exceeded those of other TFs in this study (0.017-0.082; Additional file 3 ^22^). This is consistent with the extensive interactions of this factor with many other TF cofactors in K562 cells, and its central role in specifying erythroid-specific lineage development ^3^.

We found that promoters of most TF targets contain accessible, likely functional binding sites that are significantly correlated with changes in gene expression levels. Despite a high accuracy of target recognition, sensitivity did not exceed specificity, except for IRF1 ( Table 2), due to a relatively large number of false negative genes. Promoters of non-targets are either devoid of accessible binding sites, or these sites are non-functional. In these instances, the classifier was unable to distinguish between likely functional binding sites in targets and non-functional sites in non-targets. *In vivo* co-regulation mediated by interacting cofactors, which was excluded by the classifier, assisted in distinguishing these non-functional sites that do not significantly affect gene expression ^14^.

**Figure 4.**
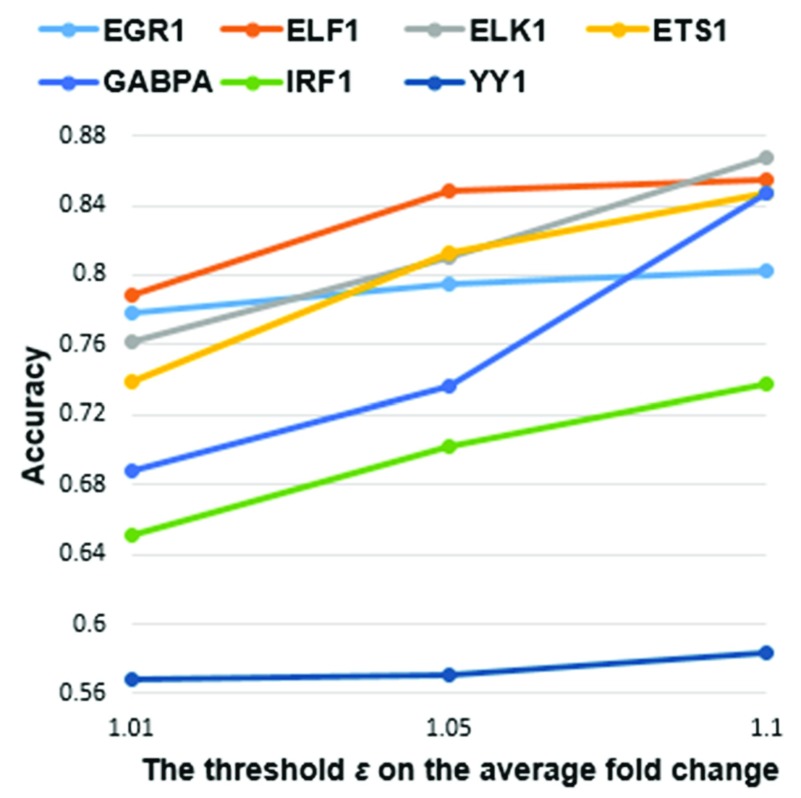
Accuracy of the Decision Tree classifier when using three different values for. Each accuracy value was averaged from 10 rounds of 10-fold cross validation. The minimum threshold *ε* of the average fold change in gene expression levels (for all guide RNAs) of the TF was determined for fold changes: 1.01, 1.05 and 1.1. The accuracy value of each individual round is indicated in Additional file 5 ^22^. As *ε* increased, accuracy for all seven TFs monotonically increased.

As the minimum differential expression threshold *ε* increased, the accuracy of the classifier for all the TFs monotonically increased, as expected ( Figure 4). In general, more significantly DE genes have been associated with a higher number of TFBSs in their promoters ^14^. Thus, at greater *ε*, there are larger differences in the values of ML features derived from TFBS clusters between direct targets and non-targets.

### Some TF target genes also display similar tissue-wide expression profiles to the TFs, themselves

To determine how many TF targets have similar tissue-wide expression profiles, we intersected the set of targets with the set of 500 PC genes with the most similar tissue-wide expression profiles for each TF ( Table 4, Additional file 6 ^22^). For example, the B lymphocyte expression profiles of TFs PAX5 and POU2F2 are similar to their respective targets, *IL21R* and *CD86*. The intersected targets for YY1 include 21 and 7 nuclear mitochondrial genes (e.g. *MRPL9* and *MRPS10*, which are subunits of mitochondrial ribosomes), respectively, in the K562 and GM19238 cell lines ^33^. YY1 is known to upregulates a large number of mitochondrial genes by complexing with PGC-1α in C2C12 cells ^34^, and genes involved in the mitochondrial respiratory chain in K562 cells ^16^. Our results are consistent with the idea that YY1 may broadly regulate mitochondrial function within all 53 tissues, in addition to the erythrocyte, lymphocyte and skeletal muscle cell lines.

**Table 4.** Intersection of TF targets and 500 protein-coding genes with the most similar tissue-wide expression profiles.

TF	Cell line	Number of targets	Size of intersection	Targets among the most similar 10 genes §
EGR1	K562	169	12	None
ELF1		78	5	None
ELK1		112	4	*GNL*1(8 ^th^)
ETS1		267	15	None
GABPA		513	25	TAF1(1 ^st^)
IRF1		457	10	None
YY1		1752	127	*MRPL9*(2 ^nd^), *BAZ1B*(6 ^th^), *ENY2*(7 ^th^), *NUB1*(8 ^th^), *USP1*(9 ^th^), *HNRNPR*(10 ^th^)
	GM19238	1040	61	*MED4*(1 ^st^), *SURF6*(3 ^rd^), *BAZ1B*(6 ^th^)
BATF		186	21	None
JUND		44	2	None
NFE2L1		58	4	None
RELA		247	13	*HMG20B*(9 ^th^)
RXRA		181	3	None
SP1		1595	81	None
TCF12		655	20	None
USF1		301	21	None
PAX5		918	86	*IL21R*(8 ^th^)
POU2F2		532	26	*CD86*(3 ^rd^)

§The rank of each target in the list of similar genes in the descending order of Bray-Curtis similarity values is shown in the brackets immediately following the target.

Between 0.4%–25% of genes with similar tissue-wide expression profiles to the TFs are actually their targets ( Table 4). The majority are non-targets whose promoters contain non-functional binding sites that lack co-regulation by corresponding cofactors. For YY1 and EGR1, we contrasted the flanking cofactor binding site distributions and strengths in the promoters of the most similarly expressed target genes (YY1: *MRPL9*, *BAZ1B*; EGR1: *CANX*, *NPM1*) with non-target genes (YY1: *ADNP*, *RNF25*; EGR1: *GUCY2F*, *AWAT1*). In these target gene promoters, strong and intermediate TFBSs recognized by SP1, KLF1, CEBPB formed heterotypic clusters with adjacent YY1 sites. Additionally, TFBSs of SP1, KLF1, and NFY tended to be present adjacent to EGR1 binding sites (Additional file 7 ^22^). These patterns contrasted with enrichment of CTCF and ETV6 binding sites in gene promoters of YY1 and EGR1 non-targets (Additional file 7 ^22^). Previous studies have reported that KLF1 is essential for terminal erythroid differentiation and maturation ^35^. Direct physical interactions between YY1 and the constitutive activator SP1 synergistically induce transcription ^36^, through activating CEBPB which promotes differentiation and suppresses proliferation of K562 cells by binding the promoter of the *G-CSFR* gene encoding a hematopoietin receptor ^37^. EGR1 and SP1 synergistically cooperate at adjacent non-overlapping sites on *EGR1* promoter but compete for binding at overlapping sites ^38^, whereas occupied CTCF binding sites often function as an insulator blocking the effects of *cis*-acting elements and preventing gene activation by mediating long-range DNA loops to alter topological chromatin structure ^39, 40^. ETV6, a member of the ETS family, is a transcriptional repressor required for bone marrow hematopoiesis and associated with leukemia development ^41^.

### Transcription factor binding site clusters buffer against expression changes from mutations in single sites

**Figure 5.**
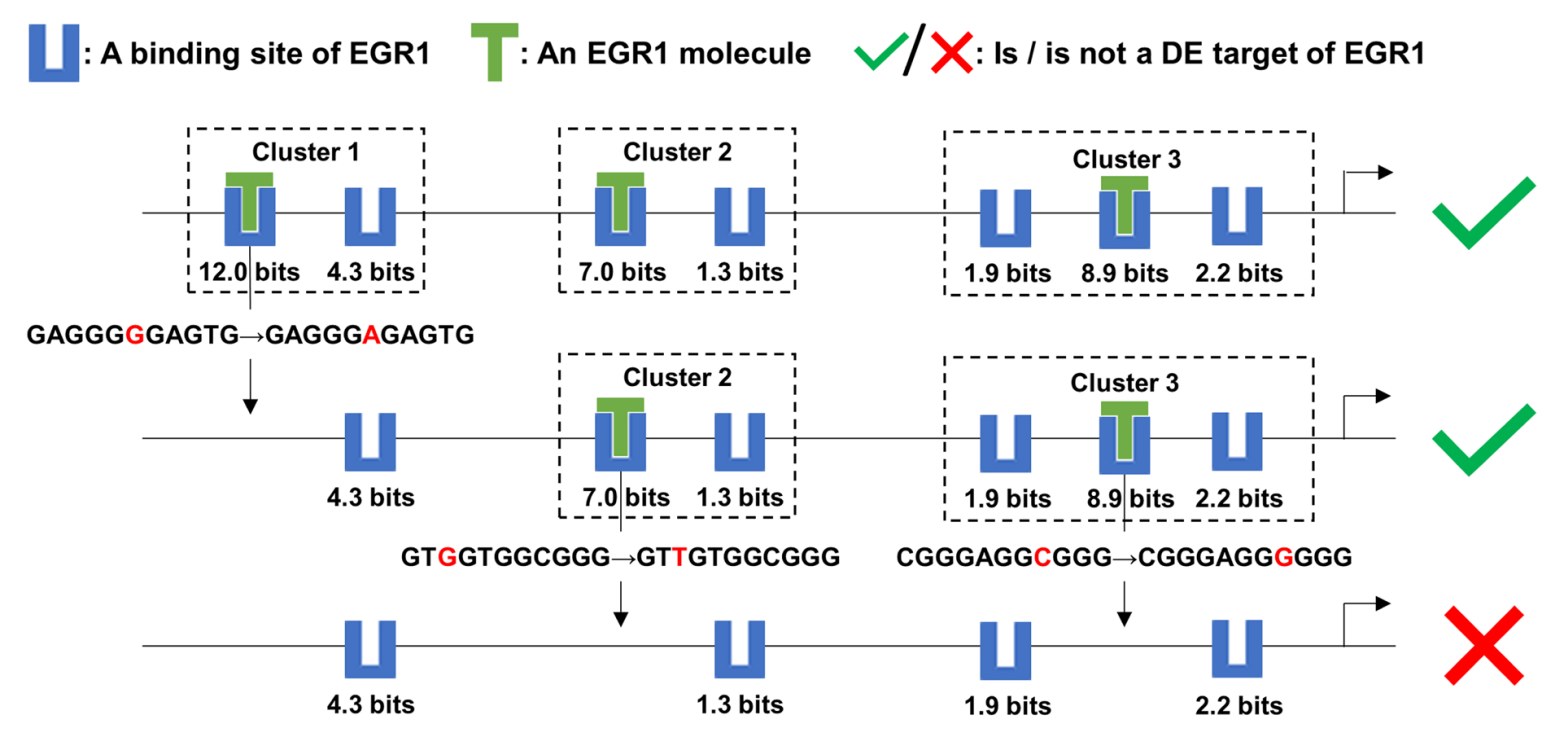
Mutation analyses on the target *MCM7* of EGR1. This figure depicts the effect of a mutation in each EGR1 binding site cluster of the *MCM7* promoter on the expression level of *MCM7*, which is a target of the TF EGR1. The strongest binding site in each cluster were abolished by a single nucleotide variant. Upon loss of all three clusters, only weak binding sites remained and EGR1 was predicted to no longer be able to effectively regulate *MCM7* expression. Multiple clusters in the promoters of TF targets confer robustness against mutations within individual binding sites that define these clusters.

These results and previous studies indicate that the promoters of direct target genes contain multiple binding site clusters. We used our ML models of TFBS organization to investigate the effects of mutations in individual binding sites on the predicted expression of TF targets. We hypothesized that alternative TF clusters in the same promoter might stabilize and compensate for the loss of a mutated TF cluster, enabling mutated promoters to retain some capacity to regulate gene transcription upon TF binding. First, we introduced artificial variants into binding sites *in silico* in the promoter of the target gene *MCM7* of EGR1 and examined the effect on the output of the ML classifier ( Figure 5). In the K562 line, *MCM7* is upregulated by EGR1. Knockdown of *MCM7* has an anti-proliferative and pro-apoptotic effect on K562 cells ^42^ and the loss of EGR1 increases leukemia initiating cells ^43^, which suggests that EGR1 may act as a tumor suppressor in K562 cells through the *MCM7* pathway.

The strongest binding site (at position chr7:100103347[hg38], - strand, *R _i_*= 12.0 bits) in the promoter was eliminated by a G->A mutation, resulting in the loss of Cluster 1 ( Figure 5), which consists of two sites (the other site at chr7:100103339, -, 4.3 bits). The other two clusters comprising weaker binding sites of intermediate strength (chr7:100102252, +, 7.0 bits; chr7:100102244, +, 1.3 bits; chr7:100101980, +, 8.9 bits; chr7:100101977, +, 2.2 bits; chr7:100101984, +, 1.9 bits) were still predicted to compensate for this mutation, so that the promoter maintains the capacity to induce *MCM7* expression ( Figure 5). Adjacent sites within the same TFBS cluster, which may individually not have sufficient affinity to strongly bind TFs and activate transcription, are capable of stabilizing binding to adjacent sites ^2^. Weaker sites can direct TF molecules to strong sites and extend the duration of physical association, termed the funnel effect ^2^. Binding stabilization between adjacent sites and the funnel effect enable CRMs comprised of information-dense clusters to be robust to mutations in individual binding sites ^2, 44^. In this example, Clusters 2 and 3 were also respectively removed by G->T and C->G mutations abolishing the strongest site in either cluster, altering the prediction of the classifier, that is, EGR1 is expected to fail to induce MCM7 transcription ( Figure 5). The remaining four sparse weak sites do not form a cluster and cannot completely supplant the disrupted strong sites.

We then examined the predicted impacts of natural SNPs on binding site strengths, clusters and predicted the regulatory state of the promoter for a direct target of each of the seven TFs from the CRISPR-generated perturbation data ( Table 5). We found that a single SNP (e.g. rs996639427 of EGR1) could affect the strengths of multiple binding sites within a cluster ( Table 5). Apart from SNPs that are predicted to abolish binding ( Figure 5), leaky variants that are expected to merely weaken TF binding are also common ( Table 5). Neither mutations that abolish TFBSs nor leaky SNPs in flanking weak binding sites would be expected to inactivate TFBS clusters (e.g. rs1030185383 and rs5874306 of IRF1), whereas SNPs with large reductions in *R _i_* values of strong sites are more likely to abolish clusters (e.g. rs865922947, rs946037930, rs917218063 and rs928017336 of YY1) ( Table 5). Multiple TF clusters enable promoters to be resilient to the effects of these mutations; only the complete inactivation of all clusters by concurrent effects of multiple SNPs within TFBSs would be capable of making a promoter to be unresponsive to TF binding (e.g. rs997328042, rs1020720126 and rs185306857 of GABPA) ( Table 5). Conversely, a small number of SNPs capable of strengthening TF binding and reinforcing the regulatory effect of the TF were also observed (e.g. rs887888062 of EGR1 and rs751263172 of ELF1) ( Table 5). In addition to deleterious mutations, potentially benign variants may also be found in promoters, consistent with the expectations of neutral theory ^45^.

**Table 5.** Mutation analyses on promoters of TF targets.

TF	Target	Normal cluster	Normal binding site ^§^	SNP ID ^§^	Variant binding site ^§^	Variant cluster ^‡^	Classifier output		
							Variant ^†^		Wild- type
EGR1 ( *R _sequence_* = 12.2899 bits)	*EID2B*	Cluster 1 of 2	***G***A ***G***GGGGC ***AT***C (chr19:39540296, -, 7.22 bits)	rs538610162 (chr19:39540296C>G)	***C***AGGGGGCATC (chr19:39540286, -, 4.84 bits)	Abolished	√	×	√
				rs759233998 (chr19:39540294C>T)	GA ***A***GGGGCATC (chr19:39540286, -, 0.06 bit)	Abolished	√		
				rs974735901 (chr19:39540288T>A)	GAGGGGGC ***T***TC (chr19:39540286, -, 6.90 bits)	Cluster 1 of 2	√		
				rs978230260 (chr19:39540287A>T)	GAGGGGGCA ***A***C (chr19:39540286, -, 5.31 bits)	Abolished	√		
		Cluster 2 of 2	***G***CGTGCGT ***G***GG (chr19:39540162, +, 1.59 bits)	rs764734511 (chr19:39540162G>A) (chr19:39540162G>C)	***A***CGTGCGTGGG (chr19:39540162, +, -0.72 bit)	Cluster 2 of 2	√		
					***C***CGTGCGTGGG (chr19:39540162, +, -0.79 bit)	Cluster 2 of 2	√		
				rs996639427 (chr19:39540170G>C)	GCGTGCGT ***C***GG (chr19:39540162, +, -5.21 bits)	Abolished	√		
			GCGT ***G***GGC ***G***C ***T*** (chr19:39540166, +, 9.72 bits)		GCGT ***C***GGCGCT (chr19:39540165, +, -0.85 bit)				
				rs1027751538 (chr19:39540174G>A)	GCGTGGGC ***A***CT (chr19:39540166, +, 5.16 bits)	Abolished	√		
				rs887888062 (chr19:39540176T>A)	GCGTGGGCGC ***A*** (chr19:39540166, +, 10.94 bits)	Cluster 2 of 2	√		
ELF1 ( *R _sequence_* = 11.2057 bits)	*HIST1H4H*	Cluster 1 of 2	GC ***GGA***AG ***CG***TG (chr6:26286540, +, 9.92 bits)	rs760968937 (chr6:26286547C>T) (chr6:26286547C>A)	GCGGAAG ***T***GTG (chr6:26286540, +, 10.71 bits)	Cluster 1 of 2	√	√	√
					GCGGAAG ***A***GTG (chr6:26286540, +, 8.84 bits)	Cluster 1 of 2	√	×	
				rs1000196206 (chr6:26286542G>C)	GC ***C***GAAGCGTG (chr6:26286540, +, -6.26 bits)	Abolished	√		
				rs144759258 (chr6:26286543G>A)	GCG ***A***AAGCGTG (chr6:26286540, +, -3.60 bits)	Abolished	√		
				rs966435996 (chr6:26286544A>G)	GCGG ***G***AGCGTG (chr6:26286540, +, 5.28 bits)	Abolished	√		
				rs950986427 (chr6:26286548G>A)	GCGGAAGC ***A***TG (chr6:26286540, +, 8.28 bits)	Cluster 1 of 2	√		
		Cluster 2 of 2	***C***AG ***GAG***ATGC ***G*** (chr6:26286483, -, 6.98 bits)	rs373649904 (chr6:26286483G>A)	***T***AGGAGATGCG (chr6:26286473, -, 0.61 bit)	Abolished	√		
				rs926919149 (chr6:26286480C>T)	CAG ***A***AGATGCG (chr6:26286473, -, -6.53 bits)	Abolished	√		
				rs751263172 (chr6:26286479T>G)	CAGG ***C***GATGCG (chr6:26286473, -, 1.24 bits)	Abolished	√		
				rs369076253 (chr6:26286473C>G)	CAGGAGATGC ***C*** (chr6:26286473, -, 6.92 bits)	Cluster 2 of 2	√		
				rs751263172 (chr6:1044474314C>T)	CAGGA ***A*** ATGCG (chr6:26286473, -, 11.43 bits)	Cluster 2 of 2	√	√	
ELK1 ( *R _sequence_* = 11.9041 bits)	*G0S2*	Cluster 1 of 2	C ***AG***GGAAG ***A***CC (chr1:209667969, -, 1.92 bits)	rs146048477 (chr1:209667961T>A)	CAGGGAAG ***T***CC (chr1:209667959, -, 2.24 bits)	Cluster 1 of 2	√	√	√
				rs887606802 (chr1:209667968T>C)	C ***G***GGGAAGACC (chr1:209667959, -, -3.35 bits)	Cluster 1 of 2	√	×	
				rs1021034916 (chr1:209667967C>T)	CA ***A***GGAAGACC (chr1:209667959, -, -3.57 bits)	Cluster 1 of 2	√		
			GAGGA ***A***ATGAG (chr1:209667969, +, 8.14 bits)	rs941962117 (chr1:209667974A>G)	GAGGA ***G***ATGAG (chr1:209667969, +, 4.11 bits)	Abolished	√		
		Cluster 2 of 2	***C***TGGAAGA ***GC***A (chr1:209673554, -, 5.91 bits)	rs896117033 (chr1:209673545G>A)	CTGGAAGAG ***T***A (chr1:209673544, -, 3.95 bits)	Cluster 2 of 2	√		
				rs971962577 (chr1:209673546C>T)	CTGGAAGA ***A***CA (chr1:209673544, -, 3.49 bits)	Cluster 2 of 2	√		
				rs1011969709 (chr1:209673554G>C)	***G***TGGAAGAGCA (chr1:209673544, -, 3.92 bits)	Abolished	√		
			CCA ***G***AAGTCA ***A*** (chr1:209673551, +, 7.44 bits)		CCA ***C***AAGTCAA (chr1:209673551, +, -5.50 bits)				
				rs1023312090 (chr1:209673561A>G)	CCAGAAGTCA ***G*** (chr1:209673551, +, 8.40 bits)	Cluster 2 of 2	√	√	
ETS1 ( *R _sequence_* = 10.0788 bits)	*TTC19*	Cluster 1 of 1	GCA ***G***GGAA ***A***GG (chr17:16022293, +, 7.92 bits)	rs1022234223 (chr17:16022296G>C)	GCA ***C***GGAAAGG (chr17:16022293, +, -4.98 bits)	Abolished	×	×	√
				rs968299415 (chr17:16022301A>T)	GCAGGGAA ***T***GG (chr17:16022293, +, 10.01 bits)	Cluster 1 of 1	√	√	
GABPA ( *R _sequence_* = 10.8567 bits)	*PLEKHB2*	Cluster 1 of 1	A ***C***A ***G***GAAAGGG (chr2:131112770, +, 10.36 bits)	rs997328042 (chr2:131112771C>T)	A ***T***AGGAAAGGG (chr2:131112770, +, -3.68 bits)	Abolished	×	×	√
				rs1020720126 (chr2:131112773G>C)	ACA ***C***GAAAGGG (chr2:131112770, +, -4.16 bits)	Abolished	×		
			T ***CT***GGAAAC ***T***A (chr2:131112760, +, 1.53 bits)	rs185306857 (chr2:131112761C>A)	T ***A***TGGAAACTA (chr2:131112760, +, -2.86 bits)	Cluster 1 of 1	√		
				rs772728699 (chr2:131112762T>A)	TC ***A***GGAAACTA (chr2:131112760, +, 5.23 bits)	Cluster 1 of 1	√		
				rs965753671 (chr2:131112769T>C)	TCTGGAAAC ***C***A (chr2:131112760, +, 2.13 bits)	Cluster 1 of 1	√		
IRF1 ( *R _sequence_* = 13.5544 bits)	*SMIM13*	Cluster 1 of 1	***GA***G ***A***A ***T***GAA ***A***GC ***A*** (chr6:11093663, +, 12.56 bits)	rs950528541 (chr6:11093663G>C)	***C***AGAATGAAAGCA (chr6:11093663, +, 8.97 bits)	Cluster 1 of 1	√	×	√
				rs886259573 (chr6:11093664A>G)	G ***G***GAATGAAAGCA (chr6:11093663, +, 9.65 bits)	Cluster 1 of 1	√		
				rs982931728 (chr6:11093666A>G)	GAG ***G***ATGAAAGCA (chr6:11093663, +, 8.09 bits)	Cluster 1 of 1	√		
				rs1020218811 (chr6:11093668T>G)	GAGAA ***G***GAAAGCA (chr6:11093663, +, 9.36 bits)	Cluster 1 of 1	√		
				rs570723026 (chr6:11093672A>G)	GAGAATGAA ***G***GCA (chr6:11093663, +, 8.01 bits)	Cluster 1 of 1	√		
				rs1004825794 (chr6:11093675A>C) (chr6:11093675A>T)	GAGAATGAAAGC ***C*** (chr6:11093663, +, 10.47 bits)	Cluster 1 of 1	√		
					GAGAATGAAAGC ***A*** (chr6:11093663, +, 10.42 bits)	Cluster 1 of 1	√		
			AA ***G***ACCAA ***AG***GCA (chr6:11093641, +, 2.43 bits)	rs1030185383 (chr6:11093649A>C)	AAGACCAA ***C***GGCA (chr6:11093641, +, -3.39 bits)	Cluster 1 of 1	√		
				rs5874306 (chr6:11093650delG)	AAGACCAAAGCAG (chr6:11093641, +, 0.90 bit)	Cluster 1 of 1	√		
				rs558896490 (chr6:11093643G>A)	AA ***A***ACCAAAGGCA (chr6:11093641, +, 7.06 bits)	Cluster 1 of 1	√	√	
YY1 ( *R _sequence_* = 12.8554 bits)	*CKLF*	Cluster 1 of 1	***G***C ***GG***C ***C***ATCGGC (chr16:66549797, -, 10.06 bits)	rs865922947 (chr16:66549791G>A)	***C***CGGCCATCGGC (chr16:66549785, -, 6.80 bits)	Cluster 1	√	×	√
				rs946037930 (chr16:66549794C>A)	GC ***T***GCCATCGGC (chr16:66549785, -, 8.02 bits)	Cluster 1	√		
				rs917218063 (chr16:66549793C>T)	GCG ***A***CCATCGGC (chr16:66549785, -, 5.41 bits)	Abolished	×		
				rs928017336 (chr16:66549791G>A)	GCGGC ***T***ATCGGC (chr16:66549785, -, -3.62 bits)	Abolished	×		
			GCCGCCCCCGTC (chr16:66549792, +, 1.34 bits)						

^§^All coordinates are based on the hg38 genome assembly. A bold italic letter in a binding site sequence indicates the base where a SNP occurs. For each normal and variant binding site sequence, the genome coordinate of its most 5’-end base and its *R _i_* value are indicated. The negative *R _i_* value of a variant binding site sequence implies this site is abolished. The SNPs strengthening binding sites and corresponding variant binding site sequences are underlined. ^‡^The impact on whether the occurrence of a single SNP resulted in the disappearance of the cluster containing it is shown; ‘Abolished’ indicates that the cluster is eliminated by the existence of the variant allele. ^†^After a single SNP occurred or multiple SNPs simultaneously occurred, the classifier produced a new prediction on whether the TF is still capable of significantly affecting gene expression via the variant promoter.

## Discussion

In this study, the Bray-Curtis similarity function was initially shown (for the *NR3C1* gene) to measure the relatedness of overall expression patterns between genes across a diverse set of tissues ( Figure 2). A ML framework distinguished similar from dissimilar genes based on the distribution, strengths and compositions of TFBS clusters in accessible promoters, which can substantially account for the corresponding gene expression patterns ( Figure 1 & Figure 3). Using gene expression knockdown data, the combinatorial use of TF binding profiles and chromatin accessibility was also demonstrated to be predictive of TF targets ( Figure 4, Table 2 & Table 3). A binding site comparison confirmed that coregulatory cofactors can be used to distinguish between functional sites in targets and non-functional ones in non-targets. Furthermore, *in silico* mutation analyses on binding sites of targets suggested that the existence of both multiple TFBSs in a cluster and multiple information-dense clusters in the same promoter enables both the cluster and the promoter to be resilient to mutations in individual TFBSs ( Figure 5, Table 5).

The DT classifier improved after intersecting promoters with DHSs in prediction of TF targets with the CRISPR-generated knockdown data ( Table 2). This intersection eliminated noisy binding sites that are inaccessible to TF proteins in promoters ^10, 31, 32^; specifically, it widened discrepancies in feature vectors between positives and negatives. If the 10 kb promoter of a gene instance does not overlap DHSs, its feature vector will only consist of 0; the percentages of negatives whose promoters do not overlap DHSs considerably exceeded those of positives (Additional file 8 ^22^), which led to an excess of negatives with feature vectors containing only 0 after intersection. This explains why these negatives are not DE targets of the TFs in the K562 and GM19238 cell lines, because their entire promoters are not open to TF molecules; other regulatory regions besides the proximal promoters (e.g. intronic enhancers ^46^) still enable the TFs to effectively control the expression of the positives with inaccessible promoters. Compared to the other six TFs, the poorer performance of the classifier on YY1 ( Table 2) is attributable to its smaller percentage of negatives with inaccessible promoters (Additional file 8 ^22^) and the larger number of functional binding sites in the K562 cell line.

Mutation analyses revealed that some deleterious TFBS mutations could be compensated for by other information-dense clusters in the same promoter ( Figure 5, Table 5) ^2, 47^; thus, predicting the effects of mutations in individual binding sites might not be sufficient to interpret downstream effects without considering their context. Though other TFBS clusters may compensate and maintain gene expression, the promoter will likely exhibit lower levels of activity than the wild-type promoter, which is a recipe for achieving natural phenotypic diversity ^44^. Few published studies in molecular diagnostics have specifically examined the effects of naturally occurring variants within clustered TFBSs. IDBC-based ML provides an alternative approach to predict deleterious mutations that impact (i.e. repress or abolish) transcription of target genes and result in abnormal phenotypes, and to minimize false positive calls of TFBS variants that alone would be expected to have little or no impact.

Apart from these TFs, the Bray-Curtis Similarity metric can be directly applied to identify the ground-truth genes with overall similar tissue-wide expression patterns to any other gene whose expression profile is known. Previous applications of this index include: a) measurement of the ecological transfer of species abundance from dense to sparse plots ^48^, b) comparative difference analyses of species quantities between reference and algorithm-derived metagenomic sample mixtures ( https://precision.fda.gov/challenges/3/view/results) and c) improvement of friend recommendations in geosocial networks by using it to compare users’ movement history ^49, 50^.

These results stimulate questions about the biological significance of genes sharing a common expression pattern, including the similarity between other regulatory regions besides proximal promoters in terms of TFBSs and epigenetic markers. This ML framework can also be applied to predict target genes for other TFs and in other cell lines, depending on the availability of corresponding knockdown data.

There are a number of limitations of our approach. The Bray-Curtis function seems unable to accurately measure the similarity between the tissue-wide expression profiles of a gene (e.g. *MIR23A*) without any detectable mRNA in any tissues and genes that are expressed in at least one tissue (e.g. ubiquitously expressed *NR3C1* and stomach-specific *PGA3*). Intuitively, *PGA3* is more similar to *MIR23A* than *NR3C1*; however, the Bray-Curtis similarity indicates that both *PGA3* and *NR3C1* are equally dissimilar to *MIR23A*. The finite number of TFs analyzed is another possible limitation in the prediction of genes with similar expression profiles. This was due to a lack of iPWMs for other TFs that were knocked down. Given that 2000-3000 sequence-specific DNA-binding TFs are estimated to be encoded in the human genome ^51^, the CRMs of many genes under concerted regulation by different TFs will likely reveal complex circuitry that is currently unknown. For example, the iPWMs for several TFs (CREB, MYB, NF1, GRF1) that bind to the *NR3C1* gene promoter to activate or repress its expression could not be successfully derived from ChIP-seq data ^3, 30^. Regarding the CRISPR-generated knockdown data, positives were inferred to be direct targets by intersecting their promoters with corresponding ChIP-seq peaks. This may not be completely accurate, due to the presence of noise peaks that do not contain true TFBSs ^3, 52, 53^. Small fold changes in the expression levels of DE targets could arise from inefficient knockdown due to suboptimal guide RNAs or to limitations of perturbing only a single allele encoding the TF ^54^. Finally, the framework presented considers only the 10 kb interval proximal to the TSS. This could not capture long range enhancer effects beyond this point; a potential way of remediating this would be to incorporate correlation-based approaches that have successfully incorporated multiple definitions of promoter length ^15^.

## Conclusions

We have developed a ML framework with a combination of information theory-based TF binding profiles of the spatial distribution and information contents of TFBS clusters, ChIP-seq and chromatin accessibility data. This framework distinguishes tissue-wide expression profiles of similar vs dissimilar genes (originally defined by the Bray Curtis function) and identified TF targets. Functional binding sites in target genes that significantly alter expression levels upon direct binding are at least partially distinguished by TF-cofactor coregulation from non-functional sites in non-targets. Finally, in-silico mutation analyses suggested that the presence of multiple information-dense clusters in a target gene promoter is capable of mitigating the effects of deleterious mutations that can significantly alter TF-regulated expression levels.

## bioRxiv

An earlier version this article is available from bioRxiv: https://doi.org/10.1101/283267 ^55^.

## Data availability

### Underlying data

The median RPKM, TSS coordinate, DNase I hypersensitivity and ChIP-seq data were respectively obtained from the GTEx Analysis V6p release ( www.gtexportal.org), Ensembl Biomart ( www.ensembl.org) and ENCODE ( www.encodeproject.org). The CRISPR- and siRNA-generated knockdown data were obtained from the supplementary information files of Dixit *et al*. ^16^ and Cusanovich *et al*. ^14^. The source datasets generated and/or analysed by this framework, along with sample results and compiled software are available from Zenodo. DOI: https://doi.org/10.5281/zenodo.1707423 ^21^.

Data are available under the terms of the Creative Commons Zero "No rights reserved" data waiver (CC0 1.0 Public domain dedication).

### Extended data

Additional files are available from Zenodo. DOI: https://doi.org/10.5281/zenodo.2611953 ^22^.

Additional file 1. The mathematical definitions of the four other similarity metrics, the workflow of the IDBC algorithm, an example feature vector, the mathematical definitions of five statistical variables to measure classifier performance, the default parameter values of classifiers in MATLAB, and histograms visualizing the first two criteria for selecting positives from the CRISPR-generated knockdown data.

Additional file 2: The lists of positives and negatives in the ML classifiers to predict genes with similar tissue-wide expression profiles to *NR3C1*.

Additional file 3: The lists of positives and negatives in the DT classifier to predict TF targets based on the CRISPR-generated knockdown data.

Additional file 4: The lists of positives and negatives in the DT classifier to predict DE direct targets based on the siRNA-generated knockdown data.

Additional file 5: The performance of the DT classifier using only TFBS counts, accuracy of each round of 10-fold cross validation, and Gini importance values of the ML features.

Additional file 6: The list of the most similar 500 PC genes to each TF in terms of tissue-wide expression profiles, and the intersection of these 500 genes and target genes of the TF.

Additional file 7: Cofactor binding sites adjacent to YY1 and EGR1 sites in the promoters of their targets and non-targets.

Additional file 8: The percentages of positives and negatives whose promoters do not overlap DHSs for the CRISPR-perturbed TFs.

Data are available under the terms of the Creative Commons Zero "No rights reserved" data waiver (CC0 1.0 Public domain dedication).

## Software availability

**Source code implementing the ML framework, including generating the figures in this article, is available at:** https://bitbucket.org/cytognomix/information-dense-transcription-factor-binding-site-clusters/.

**Archived source code at time of publication:** https://doi.org/10.5281/zenodo.1892051 ^56^.

**License:** GNU General Public License 3.0.

## References

[1] Hosseinpour B Bakhtiarizadeh MR Khosravi P: Predicting distinct organization of transcription factor binding sites on the promoter regions: a new genome-based approach to expand human embryonic stem cell regulatory network. *Gene.* 2013;531(2):212–9. 10.1016/j.gene.2013.09.011 24042128

[2] Ezer D Zabet NR Adryan B: Homotypic clusters of transcription factor binding sites: A model system for understanding the physical mechanics of gene expression. *Comput Struct Biotechnol J.* 2014;10(17):63–9. 10.1016/j.csbj.2014.07.005 25349675 PMC4204428

[3] Lu R Mucaki EJ Rogan PK: Discovery and validation of information theory-based transcription factor and cofactor binding site motifs. *Nucleic Acids Res.* 2017;45(5):e27. 10.1093/nar/gkw1036 27899659 PMC5389469

[4] Schneider TD: Information content of individual genetic sequences. *J Theor Biol.* 1997;189(4):427–41. 10.1006/jtbi.1997.0540 9446751

[5] Dinakarpandian D Raheja V Mehta S: Tandem machine learning for the identification of genes regulated by transcription factors. *BMC Bioinformatics.* 2005;6:204. 10.1186/1471-2105-6-204 16115317 PMC1208855

[6] Ouyang Z Zhou Q Wong WH: ChIP-Seq of transcription factors predicts absolute and differential gene expression in embryonic stem cells. *Proc Natl Acad Sci U S A.* 2009;106(51):21521–6. 10.1073/pnas.0904863106 19995984 PMC2789751

[7] Cheng C Alexander R Min R: Understanding transcriptional regulation by integrative analysis of transcription factor binding data. *Genome Res.* 2012;22(9):1658–67. 10.1101/gr.136838.111 22955978 PMC3431483

[8] Budden DM Hurley DG Cursons J: Predicting expression: the complementary power of histone modification and transcription factor binding data. *Epigenetics Chromatin.* 2014;7(1):36. 10.1186/1756-8935-7-36 25489339 PMC4258808

[9] Smith AD Sumazin P Xuan Z: DNA motifs in human and mouse proximal promoters predict tissue-specific expression. *Proc Natl Acad Sci U S A.* 2006;103(16):6275–80. 10.1073/pnas.0508169103 16606849 PMC1458868

[10] Zabet NR Adryan B: Estimating binding properties of transcription factors from genome-wide binding profiles. *Nucleic Acids Res.* 2015;43(1):84–94. 10.1093/nar/gku1269 25432957 PMC4288167

[11] McLeay RC Lesluyes T Cuellar Partida G: Genome-wide *in silico* prediction of gene expression. *Bioinformatics.* 2012;28(21):2789–96. 10.1093/bioinformatics/bts529 22954627 PMC3476338

[12] Karlić R Chung HR Lasserre J: Histone modification levels are predictive for gene expression. *Proc Natl Acad Sci U S A.* 2010;107(7):2926–31. 10.1073/pnas.0909344107 20133639 PMC2814872

[13] Dong X Greven MC Kundaje A: Modeling gene expression using chromatin features in various cellular contexts. *Genome Biol.* 2012;13(9):R53. 10.1186/gb-2012-13-9-r53 22950368 PMC3491397

[14] Cusanovich DA Pavlovic B Pritchard JK: The functional consequences of variation in transcription factor binding. *PLoS Genet.* 2014;10(3):e1004226. 10.1371/journal.pgen.1004226 24603674 PMC3945204

[15] Banks CJ Joshi A Michoel T: Functional transcription factor target discovery via compendia of binding and expression profiles. *Sci Rep.* 2016;6:20649. 10.1038/srep20649 26857150 PMC4746627

[16] Dixit A Parnas O Li B: Perturb-Seq: Dissecting Molecular Circuits with Scalable Single-Cell RNA Profiling of Pooled Genetic Screens. *Cell.* 2016;167(7):1853–1866.e17. 10.1016/j.cell.2016.11.038 27984732 PMC5181115

[17] Cui S Youn E Lee J: An improved systematic approach to predicting transcription factor target genes using support vector machine. *PLoS One.* 2014;9(4):e94519. 10.1371/journal.pone.0094519 24743548 PMC3990533

[18] Bray JR Curtis JT: An Ordination of the Upland Forest Communities of Southern Wisconsin. *Ecol Monogr.* 1957;27(4):325–349. 10.2307/1942268

[19] International Human Genome Sequencing Consortium: Finishing the euchromatic sequence of the human genome. *Nature.* 2004;431(7011):931–45. 10.1038/nature03001 15496913

[20] GTEx Consortium: The Genotype-Tissue Expression (GTEx) project. *Nat Genet.* 2013;45(6):580–5. 10.1038/ng.2653 23715323 PMC4010069

[21] Lu R Rogan PK: Information-dense transcription factor binding site clusters identify target genes with similar tissue-wide expression profiles and serve as a buffer against mutations - Source datasets, sample results and compiled software.2018 10.5281/zenodo.1707423 PMC6464064 31001412

[22] Lu R Rogan PK: Information-dense transcription factor binding site clusters identify target genes with similar tissue-wide expression profiles and serve as a buffer against mutations - Additional files.2018 10.5281/zenodo.2611953 PMC6464064 31001412

[23] ENCODE Project Consortium: An integrated encyclopedia of DNA elements in the human genome. *Nature.* 2012;489(7414):57–74. 10.1038/nature11247 22955616 PMC3439153

[24] Thurman RE Rynes E Humbert R: The accessible chromatin landscape of the human genome. *Nature.* 2012;489(7414):75–82. 10.1038/nature11232 22955617 PMC3721348

[25] Pearson K: Note on Regression and Inheritance in the Case of Two Parents. *Proc R Soc Lond.* 1895;58:240–2. 10.1098/rspl.1895.0041

[26] Spearman C: The Proof and Measurement of Association between Two Things. *Am J Psychol.* 1904;15(1):72–101. 10.2307/1412159 3322052

[27] He H Garcia EA: Learning from Imbalanced Data. *IEEE Trans Knowl Data Eng.* 2009;21(9):1263–1284. 10.1109/TKDE.2008.239

[28] Kent WJ Sugnet CW Furey TS: The human genome browser at UCSC. *Genome Res.* 2002;12(6):996–1006. 10.1101/gr.229102 12045153 PMC186604

[29] Sherry ST Ward MH Kholodov M: dbSNP: the NCBI database of genetic variation. *Nucleic Acids Res.* 2001;29(1):308–11. 10.1093/nar/29.1.308 11125122 PMC29783

[30] Vandevyver S Dejager L Libert C: Comprehensive overview of the structure and regulation of the glucocorticoid receptor. *Endocr Rev.* 2014;35(4):671–93. 10.1210/er.2014-1010 24937701

[31] Kaplan T Li XY Sabo PJ: Quantitative models of the mechanisms that control genome-wide patterns of transcription factor binding during early *Drosophila* development. *PLoS Genet.* 2011;7(2):e1001290. 10.1371/journal.pgen.1001290 21304941 PMC3033374

[32] Simicevic J Schmid AW Gilardoni PA: Absolute quantification of transcription factors during cellular differentiation using multiplexed targeted proteomics. *Nat Methods.* 2013;10(6):570–6. 10.1038/nmeth.2441 23584187

[33] Calvo SE Clauser KR Mootha VK: MitoCarta2.0: an updated inventory of mammalian mitochondrial proteins. *Nucleic Acids Res.* 2016;44(D1):D1251–1257. 10.1093/nar/gkv1003 26450961 PMC4702768

[34] Cunningham JT Rodgers JT Arlow DH: mTOR controls mitochondrial oxidative function through a YY1-PGC-1alpha transcriptional complex. *Nature.* 2007;450(7170):736–40. 10.1038/nature06322 18046414

[35] Tallack MR Perkins AC: KLF1 directly coordinates almost all aspects of terminal erythroid differentiation. *IUBMB Life.* 2010;62(12):886–90. 10.1002/iub.404 21190291

[36] Seto E Lewis B Shenk T: Interaction between transcription factors Sp1 and YY1. *Nature.* 1993;365(6445):462–4. 10.1038/365462a0 8003102

[37] Ferrari-Amorotti G Mariani SA Novi C: The biological effects of C/EBPalpha in K562 cells depend on the potency of the N-terminal regulatory region, not on specificity of the DNA binding domain. *J Biol Chem.* 2010;285(40):30837–50. 10.1074/jbc.M110.128272 20659895 PMC2945577

[38] Huang RP Fan Y Ni Z: Reciprocal modulation between Sp1 and Egr-1. *J Cell Biochem.* 1997;66(4):489–99. 10.1002/(SICI)1097-4644(19970915)66:4<489::AID-JCB8>3.3.CO;2-1 9282327

[39] Bell AC West AG Felsenfeld G: The protein CTCF is required for the enhancer blocking activity of vertebrate insulators. *Cell.* 1999;98(3):387–96. 10.1016/S0092-8674(00)81967-4 10458613

[40] Hou C Zhao H Tanimoto K: CTCF-dependent enhancer-blocking by alternative chromatin loop formation. *Proc Natl Acad Sci U S A.* 2008;105(51):20398–403. 10.1073/pnas.0808506106 19074263 PMC2629272

[41] Wang LC Swat W Fujiwara Y: The *TEL/ETV6* gene is required specifically for hematopoiesis in the bone marrow. *Genes Dev.* 1998;12(15):2392–402. 10.1101/gad.12.15.2392 9694803 PMC317042

[42] Tian L Liu J Xia GH: RNAi-mediated knockdown of MCM7 gene on CML cells and its therapeutic potential for leukemia. *Med Oncol.* 2017;34(2):21. 10.1007/s12032-016-0878-x 28058629

[43] Maifrede S Liebermann D Hoffman B: Egr-1, a Stress Response Transcription Factor and Myeloid Differentiation Primary Response Gene, Behaves As Tumor Suppressor in CML. *Blood.* 2014;124:2211 Reference Source

[44] Smith T Husbands P Layzell P: Fitness landscapes and evolvability. *Evol Comput.* 2002;10(1):1–34. 10.1162/106365602317301754 11911781

[45] Kimura M: The neutral theory of molecular evolution. *Sci Am.* 1979;241(5):98–100, 102, 108 passim. 504979 10.1038/scientificamerican1179-98

[46] Hural JA Kwan M Henkel G: An intron transcriptional enhancer element regulates IL-4 gene locus accessibility in mast cells. *J Immunol.* 2000;165(6):3239–49. 10.4049/jimmunol.165.6.3239 10975840

[47] Ma X Ezer D Adryan B: Canonical and single-cell Hi-C reveal distinct chromatin interaction sub-networks of mammalian transcription factors. *Genome Biol.* 2018;19(1):174. 10.1186/s13059-018-1558-2 30359306 PMC6203279

[48] Ricotta C Podaní J: On some properties of the Bray-Curtis dissimilarity and their ecological meaning. *Ecological Complexity.* 2017;31:201–205. 10.1016/j.ecocom.2017.07.003

[49] Chen X Lu R Ma X: Measuring User Similarity with Trajectory Patterns: Principles and New Metrics. *APWeb.* 2014;8709:437–448. 10.1007/978-3-319-11116-2_38

[50] Chen X Krody P Lu R: MinUS: Mining User Similarity with Trajectory Patterns. *ECML PKDD.* 2014;8726:436–439. 10.1007/978-3-662-44845-8_29

[51] Vaquerizas JM Kummerfeld SK Teichmann SA: A census of human transcription factors: function, expression and evolution. *Nat Rev Genet.* 2009;10(4):252–63. 10.1038/nrg2538 19274049

[52] Kidder BL Hu G Zhao K: ChIP-Seq: technical considerations for obtaining high-quality data. *Nat Immunol.* 2011;12(10):918–22. 10.1038/ni.2117 21934668 PMC3541830

[53] Teytelman L Thurtle DM Rine J: Highly expressed loci are vulnerable to misleading ChIP localization of multiple unrelated proteins. *Proc Natl Acad Sci U S A.* 2013;110(46):18602–7. 10.1073/pnas.1316064110 24173036 PMC3831989

[54] Shao Y Chan CY Maliyekkel A: Effect of target secondary structure on RNAi efficiency. *RNA.* 2007;13(10):1631–40. 10.1261/rna.546207 17684233 PMC1986803

[55] Lu R Rogan PK: Information-dense transcription factor binding site clusters identify target genes with similar tissue-wide expression profiles and serve as a buffer against mutations. *bioRxiv.* 2018;283267 10.1101/283267 PMC6464064 31001412

[56] Lu R Rogan PK: Information dense transcription factor binding site clusters identify target genes with similar tissue-wide expression profiles and buffer against mutations - source code. *Zenodo.* 2018 10.5281/zenodo.1892051 PMC6464064 31001412

