# Supplementary material for: Transcription factor binding site clusters identify target genes with similar tissue-wide expression and buffer against mutations
Source: F1000Res. 2019 Apr 8;7:1933. Originally published 2018 Dec 14. [Version 2] doi: 10.12688/f1000research.17363.2 (PMC6464064; doi:10.12688/f1000research.17363.2)
Supplement: Peer review history

# Peer review: Reviewer response for version 2

**DOI:** 10.5256/f1000research.20525.r46854

**Published:** 2019-04-11

**Authors:** Daphne Ezer

**Affiliations:** 1 The Alan Turing Institute for Data Science, London, UK

Most of the comments were addressed. I still have reservations about point #2 from my previous report - the authors just repeated that their approach fits the definition of a distance metric (which is true), but I think that many biologists would find it counter-intuitive that two genes with very different gene expression *patterns* over different tissues, but with the same mean expression value would be considered 'closer' than two genes that have the same "pattern" of gene expression across tissues but very different raw gene expression values. 

 However, I now suggest that the authors consider writing a follow-up paper about this, rather than address this point within this manuscript.

I have read this submission. I believe that I have an appropriate level of expertise to confirm that it is of an acceptable scientific standard.

# Peer review: Reviewer response for version 2

**DOI:** 10.5256/f1000research.20525.r46855

**Published:** 2019-04-09

**Authors:** Nicolae Radu Zabet

**Affiliations:** 1 School of Biological Sciences, University of Essex, Colchester, UK

The authors have addressed all my comments.

I have read this submission. I believe that I have an appropriate level of expertise to confirm that it is of an acceptable scientific standard.

# Peer review: Reviewer response for version 1

**DOI:** 10.5256/f1000research.18988.r42457

**Published:** 2019-02-08

**Authors:** Nicolae Radu Zabet

**Affiliations:** 1 School of Biological Sciences, University of Essex, Colchester, UK

In this manuscript, Ru and Rogan use Bray-Curtis Similarity and several machine-learning algorithms to identify genes that have similar expression patterns. They use transcription factor binding sites within promoter regions and DNA accessibility data to train their models. This is a very important question and the authors propose an interesting mechanistic approach to address it. Nevertheless, there are several limitations that need to be addressed.

 Specific comments:

  

1. While the grammar is at a good level, the way the information is presented makes the text very difficult to read. Some sentences are very long and there are many notations. One suggestion is to move some of the less important parts in the Supplementary Material.
2. On page 3 in the introduction, the authors claim that signal strength of ChIP-seq peaks are not strictly proportional to TF binding strength. This is not always true and we showed in ^1^ that in fact the number of TF molecules controls the height of the ChIP-seq peak.
3. On page 5, it is not clear why the authors talk of Features 1-3, since it seemed they had 7 features. The way the machine learning information is presented should be improved.
4. The authors test Naïve Bayes, Decision Tree, Random Forest and SVM. I was wondering if they consider also Neural Networks. They don’t need to implement that now, but they should at least mention what was behind their selection for the machine-learning algorithms.
5. One of the main findings is that DNA accessibility improves predictions, because it masks potential TF binding sites. This is something that was previously showed in the context of TF binding to the genome by us and other scientists (e.g. References 1,2,3).
6. Figure 4 needs re-plotting (e.g. x axis labels do not fit the figure).
7. In the discussion, none of the statements are referred back to any of the figures in the results section. This makes the reading difficult.
8. The lower performance for YY1 needs to be better explained. The authors claim that this could be explained by lower percentage of negatives in inaccessible promoters. Are there other examples of TFs displaying similar features? What is their performance?
9. One of the main limitations of the manuscript is that the authors use only 82 TFs and claim that there are no iPWM for others. Have they tried to use MotifDB (https://bioconductor.org/packages/release/bioc/html/MotifDb.html), which has approximately 1000 PWMs for human TFs?
10. When talking about the accuracy of the ChIP-seq signal, they could also reference this paper ^4^.

I have read this submission. I believe that I have an appropriate level of expertise to confirm that it is of an acceptable scientific standard, however I have significant reservations, as outlined above.

## References

[1] ZabetNRAdryanB: Estimating binding properties of transcription factors from genome-wide binding profiles. *Nucleic Acids Res*.2015;43(1) : 10.1093/nar/gku1269 84-94 10.1093/nar/gku1269 25432957PMC4288167

[2] KaplanTLiXYSaboPJThomasSStamatoyannopoulosJABigginMDEisenMB: Quantitative models of the mechanisms that control genome-wide patterns of transcription factor binding during early Drosophila development. *PLoS Genet*.2011;7(2) : 10.1371/journal.pgen.1001290 e1001290 10.1371/journal.pgen.1001290 21304941PMC3033374

[3] SimicevicJSchmidAWGilardoniPAZollerBRaghavSKKrierIGubelmannCLisacekFNaefFMoniatteMDeplanckeB: Absolute quantification of transcription factors during cellular differentiation using multiplexed targeted proteomics. *Nat Methods*.2013;10(6) : 10.1038/nmeth.2441 570-6 10.1038/nmeth.2441 23584187

[4] TeytelmanLThurtleDMRineJvan OudenaardenA: Highly expressed loci are vulnerable to misleading ChIP localization of multiple unrelated proteins. *Proc Natl Acad Sci U S A*.2013;110(46) : 10.1073/pnas.1316064110 18602-7 10.1073/pnas.1316064110 24173036PMC3831989

# Response

**Published:** 2019-03-25

**Authors:** Peter Rogan

**Affiliations:** University of Western Ontario, Canada

**Comment 1**: While the grammar is at a good level, the way the information is presented makes the text very difficult to read. Some sentences are very long and there are many notations. One suggestion is to move some of the less important parts in the Supplementary Material.

**Response**: The manuscript has been extensively edited to improve clarity of the presentation. Sentence lengths have been reduced. Duplicate terms and text have been eliminated. All abbreviations have been defined. Two paragraphs have been moved to the Supplementary Methods. The revised manuscript has been shortened by 400 words and approximately 2 pages.

**Comment 2**: On page 3 in the introduction, the authors claim that signal strength of ChIP-seq peaks are not strictly proportional to TF binding strength. This is not always true and we showed in ^1^ that in fact the number of TF molecules controls the height of the ChIP-seq peak.

**Response**: In (1), it was discovered that signal strengths of ChIP-seq peaks are not strictly proportional to strengths ( *R _i_* values) of the strongest TFBSs contained in the peaks. The finding in (2) provides a complementary explanation about the determinants of signal strengths of ChIP-seq peaks, which is that ‘the number of TF molecules controls the height of the ChIP-seq peak’. Therefore, this sentence is revised to “Because signal strengths of ChIP-seq peaks are not strictly proportional to strengths of the contained strongest TFBSs and are instead controlled by TFBS counts [3, 10], representing…”.

**Comment 3**: On page 5, it is not clear why the authors talk of Features 1-3, since it seemed they had 7 features. The way the machine learning information is presented should be improved.

**Response**: In this sentence, we would like to make it easier for readers to understand the generation of classifier predictors, by explaining how the seven high-level features were transformed to low-level predictors that were directly input into the classifiers. Therefore, this sentence was revised to “Each of the Features 1-3 was defined in a gene as a vector, whose size equals the number of clusters in the gene promoter; each cluster was mapped to a single value in the vector. In Features 4-7, each cluster itself was mapped to a vector corresponding to binding sites for 82 TFs (Additional file 1).” Also, Section 5 of Additional file 1 gives a specific example about the predictor vector of a gene instance.

**Comment 4**: The authors test Naïve Bayes, Decision Tree, Random Forest and SVM. I was wondering if they consider also Neural Networks. They don’t need to implement that now, but they should at least mention what was behind their selection for the machine-learning algorithms.

**Response**: We did not select Neural Networks due to two considerations. First, it requires much more data to train than traditional machine learning algorithms, as in at least thousands if not millions of labeled samples (3). In this study the numbers of both positives (i.e. protein-coding genes with similar tissue-wide expression profiles to *NR3C1*) and negatives (i.e. dissimilar genes) are 500, which is insufficient to apply Neural Networks. Second, it is more computationally expensive than traditional algorithms (4).

**Comment 5**: One of the main findings is that DNA accessibility improves predictions, because it masks potential TF binding sites. This is something that was previously showed in the context of TF binding to the genome by us and other scientists (e.g. References 1,2,3).

**Response**: Accordingly, in this revision, the last sentence of the second subsection of the Results section was revised to “Consistent with previous findings (2, 5, 6), inclusion of DHS information significantly improved AUC values of the other classifiers with the exception of Naïve Bayes.”. And in the second paragraph of the Discussion section, the second sentence was revised to “This intersection eliminated noisy binding sites that are inaccessible to TF proteins in promoters (2, 5, 6),…”

**Comment 6**: Figure 4 needs re-plotting (e.g. x axis labels do not fit the figure).

**Response**: In this revision, Figure 4 was replotted to fix this issue.

**Comment 7**: In the discussion, none of the statements are referred back to any of the figures in the results section. This makes the reading difficult.

**Response**: In the first paragraph of the Discussion section of this revision, references to the figures in the Results section were added to the following sentences, “In this study, the Bray-Curtis similarity function was initially shown (for the NR3C1 gene) to measure the relatedness of overall expression patterns between genes across a diverse set of tissues (Figure 2). A ML framework distinguished similar from dissimilar genes based on the distribution, strengths and compositions of TFBS clusters in accessible promoters, which can substantially account for the corresponding gene expression patterns (Figures 1 & 3). Using gene expression knockdown data, the combinatorial use of TF binding profiles and chromatin accessibility was also demonstrated to be predictive of TF targets (Figure 4, Tables 2 & 3). A binding site comparison confirmed that coregulatory cofactors can be used to distinguish between functional sites in targets and non-functional ones in non-targets. Furthermore, in silico mutation analyses on binding sites of targets suggested that the existence of both multiple TFBSs in a cluster and multiple information-dense clusters in the same promoter enables both the cluster and the promoter to be resilient to mutations in individual TFBS (Figure 5, Table 5).”

 In the third paragraph, references to the figures in the Results section were added to the following sentence, “Mutation analyses revealed that some deleterious TFBS mutations could be compensated for by other information-dense clusters in the same promoter (Figure 5, Table 5)”

**Comment 8**: The lower performance for YY1 needs to be better explained. The authors claim that this could be explained by lower percentage of negatives in inaccessible promoters. Are there other examples of TFs displaying similar features? What is their performance?

**Response**: In this sentence, all the seven CRISPR-perturbed TFs were split into two sets; one consisting of only YY1, the other consisting of the remaining six TFs. This sentence was comparing the performances of the Decision Tree classifiers on these two TF sets. Seen from Table 3, the classifier’s performance on YY1 was markedly lower than that on the other six TFs after intersecting promoters with DHS sites, which is due to the fact that YY1 has a smaller percentage of negatives with inaccessible promoters.

 To make this clearer, in this revision, this sentence was revised to “Compared to the other six TFs, the poorer performance of the classifier on YY1 (Table 2) is attributable to …”

**Comment 9**: One of the main limitations of the manuscript is that the authors use only 82 TFs and claim that there are no iPWM for others. Have they tried to use MotifDB (https://bioconductor.org/packages/release/bioc/html/MotifDb.html), which has approximately 1000 PWMs for human TFs?

**Response**: We selected to use these 94 iPWMs of 82 TFs that were derived from ENCODE ChIP-seq datasets using entropy minimization in (1), since we want to ensure the high quality of the iPWMs used in the analyses.

 Compared to the MotifDB PWMs, the reliability and accuracy of these iPWMs were extensively and independently validated using four methods, including detection of experimentally proven binding sites, explanation of effects of characterized SNPs, comparison with previously published motifs and statistical analyses.

 These iPWMs were used to scan for 803 experimentally confirmed TFBSs, and there was complete concordance between these true binding sites and those detected with the iPWMs, both in terms of their locations and relative strengths (1). And these iPWMs were further used to explain the experimentally observed effects of 153 SNPs on binding site strengths, based on the changes in the *R _i_* values. For 130 SNPs (∼85.0%), the predictions of the iPWMs and the experimental observations are completely concordant; for 16 SNPs (∼10.5%), the predicted and observed experimental findings are concordant, but the extents of these changes differ (e.g. TF binding is predicted to only be weakened, but binding was completely abolished in experiments); for only 7 SNPs (∼4.6%), the predicted and observed experimental changes were discordant.

 The PWMs in MotifDB are not information theory-based (i.e. not iPWMs). Instead, they are given in the form of count matrices or frequency matrices. The performance of the iPWMs that used in the present study has been shown to outperform other PWM-based approaches for binding site detection and quantification (7).

**Comment 10**: When talking about the accuracy of the ChIP-seq signal, they could also reference this paper ^4^.

**Response**: In this revision, the reference was added to the following sentence in the last paragraph of the Discussion section, “Regarding the CRISPR-generated knockdown data, positives were inferred to be direct targets by intersecting their promoters with corresponding ChIP-seq peaks. This may not be completely accurate, due to the presence of noise peaks that do not contain true TFBSs ^3, 50, 51^.”

 References:

 1. Lu,R., Mucaki,E.J. and Rogan,P.K. (2017) Discovery and validation of information theory-based transcription factor and cofactor binding site motifs. *Nucleic Acids Res.*, **45**, e27.

 2. Zabet,N.R. and Adryan,B. (2015) Estimating binding properties of transcription factors from genome-wide binding profiles. *Nucleic Acids Res.*, **43**, 84–94.

 3. Zhang,Y. and Yang,Q. (2017) A Survey on Multi-Task Learning. *ArXiv170708114 Cs*.

 4. Ghodsi,Z., Gu,T. and Garg,S. (2017) SafetyNets: Verifiable execution of deep neural networks on an untrusted cloud. In *Advances in Neural Information Processing Systems*.Vol. 2017-December, pp. 4673–4682.

 5. Kaplan,T., Li,X.-Y., Sabo,P.J., Thomas,S., Stamatoyannopoulos,J.A., Biggin,M.D. and Eisen,M.B. (2011) Quantitative models of the mechanisms that control genome-wide patterns of transcription factor binding during early Drosophila development. *PLoS Genet.*, **7**, e1001290.

 6. Simicevic,J., Schmid,A.W., Gilardoni,P.A., Zoller,B., Raghav,S.K., Krier,I., Gubelmann,C., Lisacek,F., Naef,F., Moniatte,M., *et al.* (2013) Absolute quantification of transcription factors during cellular differentiation using multiplexed targeted proteomics. *Nat. Methods*, **10**, 570–576.

 7. Erill I and O’Neill MC. (2009) A reexamination of information theory-based methods for DNA binding site identification. BMC Bioinformatics, **10**, 57.

# Peer review: Reviewer response for version 1

**DOI:** 10.5256/f1000research.18988.r42458

**Published:** 2019-01-09

**Authors:** Daphne Ezer

**Affiliations:** 1 The Alan Turing Institute for Data Science, London, UK

I think that this is an interesting paper that should be published. Often, gene expression patterns are clustered and TF binding sites are used as features to build a classifier for identifying the cluster to which those genes belong or similar schemes such as clustering TFs and gene expression together- see Clements et al ^1^ and Berman et al ^2^. However, a biologist may want to identify what TFs regulate a specific gene of interest. They could then use the 'Bray-Curtis Similarity' index to find a set of genes whose expression pattern is similar to their gene of interest.  Then, they can use the pipeline presented here to identify features that are predictive of this kind of gene expression pattern.  

 They also create a scheme to test how different combinations of features from different experiments influence their predictions.

 I think that the text would garner much more interest if it focused more on the research questions that are being addressed. The method details are discussed in depth, but the big picture is hard to find amidst the details.  

**Main points:**

1. One of the main things that bothers me about the Bray-Curtis Similarity metric is that it seems to assume that tissues are independently sampled. However. we see from Fig.1 that there are many brain samples that seem to (at least in the three genes shown) have similar gene expression values. Is there a lot of covariance between gene expression values in pairs of tissues? If so, is there a way to adjust this metric to acknowledge stratification of the tissues. I don't think that the whole paper needs to be re-written with a new metric, but it would be nice if the authors address this directly.
2. Another issue I have is with this metric is that it uses RPKM gene expression values in the Bray-Curtis Similarity metric. Imagine that two genes have extremely high gene expression values in some tissue (like the brain) and low values in another tissue (like the pancreas). However, one of these genes is always expressed at 10 times the level of the other gene. Lets say that a third gene has almost no change of gene expression value across the tissues but has a mean RPKM that is similar to the first gene's mean RPKM. Would the Bray-Curtis SImilarity metric say that gene 1 and 2 are more similar? Or gene 1 and 3? If gene 1 and 3, then this might be resolved by comparing z-scores or otherwise normalising gene expression values across tissues.
3. Every time a machine learning method is named, it should be clear: (1) what are the input features (ii) what are the labels -- i.e. what is being classified (iii) what is the cross-validation or training-testing-validation scheme. Since there are so many machine learning things being done, it is hard sometimes to make sense of what is being done in each specific case.  
4. For the method described in (B) in Fig 1: Does it necessarily make sense to compare the 'most similar' to the 'least similar'? Genes that have exactly the opposite gene expression pattern to the one you are interested might be tightly regulated by a different set of TFs. You might be picking up this signal instead of the one you care about! This might be an even bigger issue since you are using raw gene expression values-- genes that are very highly or very lowly expressed in all tissues might always come up in your negative set.
5. Biologists don't just want good classifiers, they want feature selection! Can you show the Gini scores of the features in a supplemental table?

**Smaller changes:**

1. Is all the code for generating every figure available online? Let's help make research reproducible.
2. If you have 10 values (from cross validation), why don't you show them all in Figure 4 so we can evaluate the spread.
3. "Our *in-silico* mutation analyses revealed that some deleterious TFBS mutations could be compensated for by other information-dense clusters in the same promoter ^2^; thus, predicting the effects of mutations in individual binding sites might not be sufficient to interpret downstream effects without considering their context." This is something that me and my collaborators have recently studied ^3^. Don't feel pressure to add this citation-- I just thought it would be interesting for you to read! (Also, thanks for discussing IDBC in this paper-- I hadn't heard of it before but it would be relevant to my research.)
4. It would be great if you discussed how Bray-Curtis is used in other fields in the Discussion.  
5. Better subsection names in the results section - emphasizing the biological conclusions rather than what was done.

I have read this submission. I believe that I have an appropriate level of expertise to confirm that it is of an acceptable scientific standard, however I have significant reservations, as outlined above.

## References

[1] ClementsMvan SomerenEPKnijnenburgTAReindersMJ: Integration of known transcription factor binding site information and gene expression data to advance from co-expression to co-regulation. *Genomics Proteomics Bioinformatics*.2007;5(2) : 10.1016/S1672-0229(07)60019-9 86-101 10.1016/S1672-0229(07)60019-9 17893074PMC5054099

[2] BermanBPNibuYPfeifferBDTomancakPCelnikerSELevineMRubinGMEisenMB: Exploiting transcription factor binding site clustering to identify cis-regulatory modules involved in pattern formation in the Drosophila genome. *Proc Natl Acad Sci U S A*.2002;99(2) : 10.1073/pnas.231608898 757-62 10.1073/pnas.231608898 11805330PMC117378

[3] MaXEzerDAdryanBStevensT: Canonical and single-cell Hi-C reveal distinct chromatin interaction sub-networks of mammalian transcription factors. *Genome Biology*.2018;19(1) : 10.1186/s13059-018-1558-2 10.1186/s13059-018-1558-2 30359306PMC6203279

# Response

**Published:** 2019-03-25

**Authors:** Peter Rogan

**Affiliations:** University of Western Ontario, Canada

**Comment 1**: One of the main things that bothers me about the Bray-Curtis Similarity metric is that it seems to assume that tissues are independently sampled. However. we see from Fig.1 that there are many brain samples that seem to (at least in the three genes shown) have similar gene expression values. Is there a lot of covariance between gene expression values in pairs of tissues? If so, is there a way to adjust this metric to acknowledge stratification of the tissues. I don't think that the whole paper needs to be re-written with a new metric, but it would be nice if the authors address this directly.

**Response**: ① There are 13 brain tissues among all the 53 tissues investigated by GTEx. Admittedly, genes tend to exhibit closer expression values between some of these brain tissues; for example, seen from Figure 2, the *NR3C1* gene has close, low expression values in multiple brain tissues (Amygdala, Anterior cingulate cortex (BA24), Caudate (basal ganglia), etc).

 To investigate how much this covariance can influence similarity values between tissue-wide expression profiles of genes computed by the Bray-Curtis function, using the brain tissues as an example, we retained only one brain tissue and removed all other brain tissues at one time, and recomputed the Bray-Curtis similarity values between *NR3C1* and all other protein-coding genes. Thus, there are 13 variant cases due to the presence of 13 brain tissues. Then we compared the resultant set of 500 most similar genes in each variant case to that when all 53 tissues were used (given in Additional file 2).

 All of the top 100 most similar genes when using all 53 tissues were among the top 500 genes in every variant case. The top 200 genes when using all 53 tissues differed by 0-3 genes from the top 500 genes in variant cases. The top 500 genes when using all 53 tissues differed by approximately 22% (112-117) from top 500 genes in variant cases. This suggests that the increased number of brain tissues does not significantly influence the results of the Bray-Curtis metric for the most similar genes but does affect results at lower similarity threshold.

 Especially, in all 14 cases (i.e. the 13 variant cases and using all 53 tissues), the three most similar genes to *NR3C1* are the same ( *SLC25A32*, *TANK*, *CDC27*). Therefore, this covariance between these brain tissues is not a dominant factor in identifying genes with similar tissue-wide expression profiles to a particular gene using the Bray-Curtis Function.

 ② On the other hand, the situation is also present that a gene has closer expression values between two tissues from two different organs, than between two more similar tissues from the same organ. For example, both the Cerebellar Hemisphere (CH) tissue and the Amygdala tissue are from the brain; the Visceral Adipose (VA) tissue and the Adrenal Gland (AG) tissue are not. Seen from Figure 2, for the *NR3C1* gene’s expression, there is a larger difference between CH and Amygdala. Instead, CH is closer to VA, whereas Amygdala is closer to AG.

 Despite well established developmental lineages for these tissues, we prefer not to make assumptions regarding the covariance in expression values between similar tissues from the same organ. The null hypothesis should not discriminate between tissues or weight them differently, without explicit prior information about tissue-specific expression, which is what we are trying to measure. For this reason, when computing similarity values between tissue-wide expression profiles of genes using the Bray-Curtis Function or other metrics, it may be more appropriate to assign the same weight to every tissue and treat every tissue equally.

**Comment 2**: Another issue I have is with this metric is that it uses RPKM gene expression values in the Bray-Curtis Similarity metric. Imagine that two genes have extremely high gene expression values in some tissue (like the brain) and low values in another tissue (like the pancreas). However, one of these genes is always expressed at 10 times the level of the other gene. Lets say that a third gene has almost no change of gene expression value across the tissues but has a mean RPKM that is similar to the first gene's mean RPKM. Would the Bray-Curtis SImilarity metric say that gene 1 and 2 are more similar? Or gene 1 and 3? If gene 1 and 3, then this might be resolved by comparing z-scores or otherwise normalising gene expression values across tissues.

**Response**:

 Gene 1 and 3 will be more similar according to Bray-Curtis Similarity. The inference is as follows:

 Assume that there are two tissues *t _1_* and *t _2_*. The expression values of Gene 1 in the two tissues are [a, b] (b>>a>0), the expression values of Gene 2 are [10a, 10b], and the expression values of Gene 3 are [(a+b)/2, (a+b)/2].

 Then the Bray-Curtis similarity value between Gene 1 and Gene 2 is:

*sim _BC_ (G1,G2) = *2/11. 

 The Bray-Curtis similarity value between Gene 1 and Gene 3 is:

*sim _BC_ (G1,G3) = *(3a+b)/(2a+2b).

 Thus, *sim _BC_ (G1,G3) *> *sim _BC_ (G1,G2).*

 However, this is not unreasonable. In other words, this is not a problem, thus it does not need to be resolved. There is no ground-truth relationship between *sim _BC_ (G1,G2)* and *sim _BC_ (G1,G3)*. The reason is described below.

 When measuring the similarity between two vectors, there are two factors to be considered: 1. the sizes of the vectors (i.e. the distance between the two vectors), 2. the directions of the vectors (i.e. the angle between the two vectors). In this context of measuring similarity between tissue-wide expression values of genes (each gene is mapped to a vector), both factors matter.

 It is stated in the Methods section that Bray-Curtis Similarity satisfies three desirable properties. In fact, Property 2 (achieving the maximal similarity value 1 if and only if two vectors are identical) ensures Factor 1 to be considered, and Property 3 (larger values having a larger impact on the resultant similarity value) ensures Factor 2 to be considered.

 Thus, Table 1 shows that Bray-Curtis Similarity is more appropriate than the other five metrics, which is exactly due to the fact it takes both factors into account. In contrast, Euclidean Similarity does not take vectors’ directions into account; Cosine Similarity, Pearson Correlation and Spearman Correlation do not take the sizes of the vectors into account.

 Thus, to be able to infer a ground-truth similarity relationship, on both Factor 1 and Factor 2 the intuitive comparison results must be concordant. In Example 1 (see Additional File 1), the angle between Gene *A* and Gene *C* is identical to that between Gene *B* and Gene *C*, and the distance between *A* and *C* is larger than that between *B* and *C*; thus, *sim (A,C) < sim (B,C)*. Similarly, the distance between *A* and *D* is identical to that between *E* and *F*, and the angle between *A* and *D* is larger than that between *E* and *F*; thus, *sim(A,D) < sim(E,F)*.

 But in this case, the distance between Gene 1 and Gene 2, i.e. 9*sqrt( *a ^2^ + b ^2^*),  is larger than that between Gene 1 and Gene 3, i.e. ( *b-a*)/sqrt(2), but the angle between Gene 1 and Gene 2 (i.e. 0) is smaller than that between Gene 1 and Gene 3 (>0). Thus, a ground-truth similarity relationship is unable to be inferred.

 Thus, the result given by Bray-Curtis Similarity, i.e. *sim _BC _(G1,G3*) > *sim _BC_ (G1,G2), * is not unreasonable.

**Comment 3**: Every time a machine learning method is named, it should be clear: (1) what are the input features (ii) what are the labels -- i.e. what is being classified (iii) what is the cross-validation or training-testing-validation scheme. Since there are so many machine learning things being done, it is hard sometimes to make sense of what is being done in each specific case.

**Response**: In the module to predict genes with similar tissue-wide expression profiles to a particular gene, to make these points clearer, the following changes were made:

 (i) Using the red color Figure 1A shows that seven features were derived from TFBS clusters. In addition, in the legend of Figure 1A, the following sentence was added “The seven ML features derived from TFBS clusters were described in the Methods section.” The second paragraph of the second subsection of the Methods section details the seven features; its first sentence was revised to “The seven information density-related ML features derived from each TFBS cluster included …”

 (ii) The first sentence of the ‘Prediction of genes with similar tissue-wide expression profiles” subsection of the Methods section was revised’ to ‘The framework for predicting whether the tissue-wide expression profile of a gene resembles a particular gene is indicated in Figure 1A, B.”, so that it is clear that the two labels are ‘similar to the particular gene’ and ‘dissimilar to the particular gene’. In addition, Figure 1B also indicates that 500 most similar genes and 500 most dissimilar genes were selected as positives and negatives.

 (iii) The last step (‘Performance evaluation’) of Figure 1A was revised to ‘Performance evaluation (ROC curve/10-fold cross validation)’; the red color indicates that ROC curves were used to validate the classifiers in this module. In addition, the last sentence of the legend of Figure 1A was revised to “The performance of ML classifiers was evaluated with ROC curves and 10-fold cross validation”.

 The last sentence of the second subsection of the Methods section was revised to “This allowed all genes to be used as training data for ML classifiers. Default parameter values for these classifiers were used to generate ROC curves in MATLAB”, and also the first sentence of the corresponding second subsection of the Results section was revised to “Several ML classifiers (Naïve Bayes, Decision Tree (DT), Random Forest and Support Vector Machines (SVM) with four different kernels) were evaluated to determine how well TFBS-related features could predict genes with tissue-wide expression profiles similar to NR3C1. Their performance were compared using ROC curves…”. Thus, it is now clear that ROC curves were used to validate the classifiers and all instances were used as training data (i.e. there were no test sets).

 In the module to predict TF target genes, to make these points clearer, the following changes were made:

 (i) Using the blue color Figure 1A shows that six features were derived from TFBS clusters. Also, the penultimate sentence of the last paragraph of the “Using gene expression in the CRISPR-based perturbations” subsection of the Methods section states “Six features (Features 1-5 and 7) were derived from each homotypic cluster (i.e. Feature 6 became identical to Feature 3, because only binding sites from a single TF were used) (Figure 1A).”. Combining with the detailed descriptions about what the features are in the second paragraph of the previous subsection, it is clear that these six features (Features 1-5 and 7) were used.

 (ii) The last sentence of the first paragraph of the ‘Using gene expression in the CRISPR-based perturbations” subsection of the Methods section was revised to “We defined a positive TF target gene in a cell line as:…” And the sentence in the fifth paragraph was revised to “If the coefficients of all guide RNAs of the TF for a gene are zero, the gene was defined as a negative (i.e. a non-target gene).” Thus it is clear that the two labels are “TF target genes” and “non-target genes”. Combining with the last sentence of the first paragraph of the Methods section, ‘Since protein-coding (PC) sequences represent the most widely studied and best understood component of the human genome [19], positives and negatives for deriving machine learning classifiers for predicting DE direct TF target genes that encode proteins (TF targets, below) were obtained from CRISPR- and siRNA-generated knockdown data’, it is clear that the ‘target genes’ here stands for ‘PC, direct, DE target genes’.

 (iii) As stated above, the last step (‘Performance evaluation’) of Figure 1A was revised to ‘Performance evaluation (ROC curve/10-fold cross validation)’; the blue color indicates that 10- fold cross validations were used to validate the classifiers in this module. In addition, the last sentence of the legend of Figure 1A was revised to “The performance of ML classifiers was evaluated with ROC curves and 10-fold cross validation”. The last sentence of the last paragraph of the “Using gene expression in the CRISPR-based perturbations” subsection of the Methods section was revised to “The results of 10 rounds of 10-fold cross validation were averaged to evaluate the accuracy of the classifier.” Thus it is clear that the validation scheme is 10-fold cross validation.

**Comment 4**: For the method described in (B) in Fig 1: Does it necessarily make sense to compare the 'most similar' to the 'least similar'? Genes that have exactly the opposite gene expression pattern to the one you are interested might be tightly regulated by a different set of TFs. You might be picking up this signal instead of the one you care about! This might be an even bigger issue since you are using raw gene expression values-- genes that are very highly or very lowly expressed in all tissues might always come up in your negative set.:

**Response**: Yes, it makes sense. As stated in the above response to Comment 3, in this module to predict genes with similar tissue-wide expression profiles to a particular gene, the two labels are ‘similar to the given gene’ and ‘dissimilar to the given gene’. Therefore, it makes the most sense that the most similar genes were selected as positives and the most dissimilar genes were selected as negatives.

 The first sentence of the last paragraph of the Background section, “…the distribution and composition of *cis*-regulatory modules in promoters substantially determines gene expression profiles…, is exactly the underlying rationale why this machine learning framework is able to distinguish between “similar genes” and “dissimilar genes”. In other words, “similar genes” and “dissimilar genes” have different expression patterns, presumably because they have different organizations and compositions (i.e. different TF sets) of TFBSs in their promoters. Therefore, the potential fact that “similar genes” and “dissimilar genes” are regulated by different TF sets was exactly what we expected to see and validate.

**Comment 5**: Biologists don't just want good classifiers, they want feature selection! Can you show the Gini scores of the features in a supplemental table?:

**Response**: In the module to predict TF target genes based on CRISPR- and siRNA-generated knockdown data, to assess the relative importance of the six features to the Decision Tree classifiers, we computed their Gini importance values, which are added to Additional file 5.

 For the seven CRISPR-perturbed TFs in the K562 cell line, the cluster-level Features 1-3, especially Feature 3 capturing the information density of TFBS clusters, have the largest contribution to the classifiers’ predictive power. By contrast, for the 11 siRNA-perturbed TFs in the GM19238 cell line, the binding site-level Feature 5 capturing the spatial distribution of strong TFBSs has the largest contribution.

 Accordingly, in this revision, this observation is described in the second and third sentences of the first paragraph of the third subsection of the Results section.

**Comment 6**: Is all the code for generating every figure available online? Let's help make research reproducible.

**Response**: As stated in the Software availability section, the code that implemented this machine learning framework and produced all the results has been deposited at https://bitbucket.org/cytognomix/information-dense-transcription-factor-binding-site-clusters/src and https://doi.org/10.5281/zenodo.1892051. The input of the code to derive the figures is directly taken from the output of the ML framework code. There are no intermediate steps or variable required to generate the figures in MATLAB.  

 The code for generating the figures is used to visualize the results. in this revision, we also deposited the MATLAB code for generating all the figures at https://bitbucket.org/cytognomix/information-dense-transcription-factor-binding-site-clusters/src

**Comment 7**: If you have 10 values (from cross validation), why don't you show them all in Figure 4 so we can evaluate the spread.

**Response**: To avoid making Figure 4 too complicated, in this revision, the accuracy values of all individual rounds of 10-fold cross validations in prediction of TF target genes were added to Additional file 5. Accordingly, the legends of Table 2, Figure 4 and Table 3 were revised to indicate this.

**Comment 8**: "Our in-silico mutation analyses revealed that some deleterious TFBS mutations could be compensated for by other information-dense clusters in the same promoter(2); thus, predicting the effects of mutations in individual binding sites might not be sufficient to interpret downstream effects without considering their context." This is something that me and my collaborators have recently studied3. Don't feel pressure to add this citation-- I just thought it would be interesting for you to read! (Also, thanks for discussing IDBC in this paper-- I hadn't heard of it before but it would be relevant to my research.)

**Response**: In this revision, this publication has now been referenced (number 47) in the manuscript.

**Comment 9**: It would be great if you discussed how Bray-Curtis is used in other fields in the Discussion.

**Response**:The following sentences were added to the penultimate paragraph, “Previous applications of this index include: a) measurement of the ecological transfer of species abundance from dense to sparse plots ^48^ and comparative difference analyses of species quantities between reference and algorithm-derived metagenomic sample mixtures ( https://precision.fda.gov/challenges/3/view/results). b) improvement of friend recommendation in geosocial networks by using it to compare users’ movement history ^49, 50^ ..”

**Comment 10**: Better subsection names in the results section - emphasizing the biological conclusions rather than what was done.

**Response**: In the Results section of this revision, the title of each subsection was revised as follows, now summarizing the primary conclusion from this subsection.

 The title of the first subsection was revised from ‘Similarity between GTEx tissue-wide expression profiles of genes’ to ‘The Bray-Curtis Function can accurately quantify the similarity between tissue-wide gene expression profiles’.

 The title of the second subsection was revised from ‘Prediction of genes with similar GTEx tissue-wide expression profiles’ to ‘The Decision Tree classifier performed best in prediction of genes with similar tissue-wide expression profiles’.

 The title of the third subsection was revised from ‘Prediction of TF targets” to ‘The Decision Tree classifier was predictive of TF target genes’.

 The title of the fourth subsection was revised from ‘Intersection of genes with similar tissue-wide expression profiles and TF targets’ to ‘Some TF target genes also display similar tissue-wide expression profiles to the TFs, themselves.

 The title of the fourth subsection was revised from ‘Mutation analyses on promoters of direct targets’ to ‘Transcription factor binding site clusters buffer against expression changes from mutations in single sites’.
